# Periostin Contributes to Immunoglobulin a Nephropathy by Promoting the Proliferation of Mesangial Cells: A Weighted Gene Correlation Network Analysis

**DOI:** 10.3389/fgene.2020.595757

**Published:** 2021-01-07

**Authors:** Jingkui Wu, Qisheng Lin, Shu Li, Xinghua Shao, Xuying Zhu, Minfang Zhang, Wenyan Zhou, Zhaohui Ni

**Affiliations:** Department of Nephrology, School of Medicine, Renji Hospital, Shanghai Jiao Tong University, Shanghai, China

**Keywords:** weighted gene correlation network analysis, immunoglobulin a nephropathy, POSTN, proliferation, mesangial cells

## Abstract

Immunoglobulin A nephropathy (IgAN) is a known cause of end-stage kidney disease, but the pathogenesis and factors affecting prognosis are not fully understood. In the present study, we carried out weighted gene correlation network analysis (WGCNA) to identify hub genes related to the occurrence of IgAN and validated candidate genes in experiments using mouse mesangial cells (MMCs) and clinical specimens (kidney tissue from IgAN patients and healthy controls). We screened the GSE37460 and GSE104948 differentially expressed genes common to both datasets and identified periostin (*POSTN*) as one of the five key genes using the cytoHubba plugin of Cytoscape software and by receiver-operating characteristic curve analysis. The top 25% of genes in the GSE93798 dataset showing variable expression between IgAN and healthy tissue were assessed by WGCNA. The royalblue module in WGCNA was closely related to creatinine and estimated glomerular filtration rate (eGFR) in IgAN patients. *POSTN* had very high module membership and gene significance values for creatinine (0.82 and 0.66, respectively) and eGFR (0.82 and −0.67, respectively), indicating that it is a co-hub gene. In MMCs, *POSTN* was upregulated by transforming growth factor β1, and stimulation of MMCs with recombinant POSTN protein resulted in an increase in the level of proliferating cell nuclear antigen (PCNA) and a decrease in that of B cell lymphoma-associated X protein, which were accompanied by enhanced MMC proliferation. *POSTN* gene knockdown had the opposite effects. Immunohistochemical analysis of kidney tissue specimens showed that POSTN and PCNA levels were elevated, whereas the rate of apoptosis was reduced in IgAN patients relative to healthy controls. POSTN level in the kidney tissue of IgAN patients was positively correlated with creatinine level and negatively correlated with eGFR. Thus, POSTN promotes the proliferation of MCs to promote renal dysfunction in IgAN.

## Introduction

Immunoglobulin A nephropathy (IgAN) is a primary glomerulonephritis accounting for 30–40% of biopsy-confirmed cases of glomerulonephritis worldwide. Although the disease course varies widely, it can lead to end-stage renal disease within 10–20 years after onset (Manno et al., 2007). IgA-dominant deposition in the mesangial region of the glomerulus is a key factor in the occurrence of IgAN; however, the etiology and pathogenesis of IgAN are poorly understood (Lin et al., 2014; Roberts, 2014).

Renal injury in IgAN is caused by accumulation of aggregated immune complexes in the glomerular membrane, including IgA1 and/or IgA1–IgG, which results in excessive synthesis of extracellular matrix (ECM) (Cox et al., 2010). As changes in gene expression can precede histologic damage, analysis of the kidney transcriptome can reveal gene signatures related to the development of kidney injury (Schena et al., 2018). In isolated glomeruli or tubulointerstitial tissue from renal biopsy specimens of patients with IgAN, the expression of proteoglycans directly involved in kidney injury was increased, suggesting their clinical utility as prognostic biomarkers; moreover, 11 transcriptional events related to proteinuria have been identified in tubulointerstitial cells (Reich et al., 2010; Ebefors et al., 2011). However, the detailed mechanism by which these events lead to IgAN has not been reported.

Weighted gene correlation network analysis (WGCNA) is a powerful bioinformatics approach in which a coexpression network is established based on a gene expression dataset. An important feature of WGCNA is that it can be used to reconstruct a gene coexpression module that is represented by key genes (Langfelder and Horvath, 2008). Applying WGCNA to the investigation of IgAN can provide insight into the pathogenesis of this disease by revealing the relationship between key genes and clinical features.

To this end, in the present study, we examined differentially expressed genes (DEGs) in IgAN using the GSE37460 and GSE104948 datasets, which comprise gene expression data from the glomeruli of patients with IgAN. Key genes were identified by combining cytoHubba—a plugin for the open-source Cytoscape software (Shannon et al., 2003)—and receiver-operating characteristic (ROC) curve analysis. We used WGCNA to build a network for the GSE93798 dataset and incorporated clinical features to identify modules related to renal function [creatinine and estimated glomerular filtration rate (eGFR)]. Finally, we examined the functions of the key genes in mesangial cells (MCs) and used clinical specimens to further analyze the relationship between these gene and renal function in IgAN.

## Materials and Methods

### Study Design

**Figure 1:**
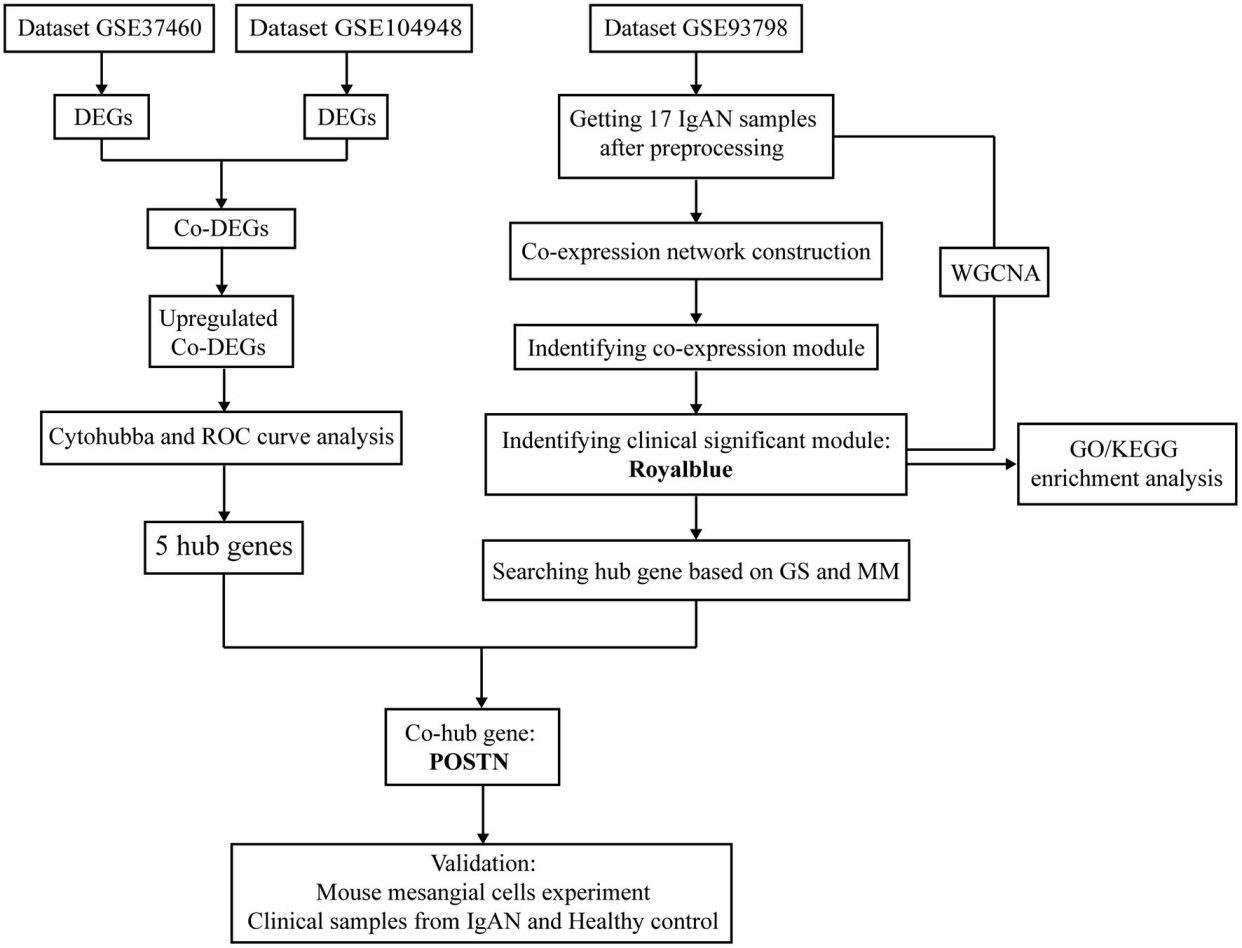
Flow diagram of the study including data processing, analysis and experimental verification.

A flow diagram of the study is shown in Figure 1. Briefly, we first analyzed the GSE37460 and GSE104948 and identified DEGs common to the two datasets. Hub genes were identified by examining the upregulated DEGs using the cytoHubba plugin of Cytoscape and by ROC curve analysis. Genes expressed in IgAN patients in the GSE93798 dataset were selected for WGCNA to identify key modules related to renal function (creatinine and eGFR); the associated hub genes were identified based on gene significance (GS) and module membership (MM) values. Co-hub genes with potential value for further analysis were also extracted. We then performed *in vitro* and *in vivo* experiments using mouse (M)MCs and clinical specimens, respectively, to validate the relevance of the co-hub genes to IgAN. This study was approved by the Renji Hospital Ethics Committee of Shanghai Jiaotong University School of Medicine (approval no. KY[2019]002).

### Dataset Information and Screening for Overlapping DEGs

The GSE37460 (Berthier et al., 2012), GSE104948 (Grayson et al., 2018), and GSE93798 (Liu et al., 2017) Series Matrix datasets were obtained by microarrays from the Gene Expression Omnibus (GEO; http://www.ncbi.nlm.nih.gov/geo/). The datasets were generated from renal biopsy specimens from IgAN patients and healthy control subjects (Table 1).

**Table 1:** Datasets used in this study^*^.

**Serial number**	**Tissue**	**Platform**	**Original probes**	**IgAN**	**Healthy control**
GSE37460	Glomeruli	GPL14663	12,020	27	9
GSE104948	Glomeruli	GPL24120	12,074	27	3
GSE93798	Glomeruli	GPL22945	19,702	20	22

* These datasets are derived from kidney tissue samples of IgAN patients and healthy controls.

Differential gene expression analysis and preprocessing by normalization of the GSE37460 and GSE104948 datasets were carried out using R v4.0.0 software with the limma package (http://www.bioconductor.org/packages/release/bioc/html/limma.html). The *P*-value of genes in these datasets was defined as an adjusted *P*-value. After analysis, an adjusted *P* < 0.05 and |log2(fold change)| ≥1 were selected as the cutoffs for screening DEGs. A volcano plot and hierarchical clustering heatmap were used to visualize the differential expression of all genes and top 100 DEGs, respectively. DEGs present in both datasets were considered overlapping DEGs, and those that were upregulated were retained for further analysis (http://bioinformatics.psb.ugent.be/webtools/Venn/).

### Construction of a Protein–Protein Interaction Network and Identification of Hub Genes

We used the Search Tool for the Retrieval of Interacting Genes/Proteins (STRING, http://string-db.org/) (Szklarczyk et al., 2015) to construct a protein–protein interaction (PPI) network of the identified DEGs. The network was analyzed using the cytoHubba plugin of Cytoscape (http://apps.cytoscape.org/apps/cytohubba), which includes 12 algorithms for filtering hub genes from PPI modules. The stress and betweenness (Chin et al., 2014; Thakur et al., 2017) statistical measures were used to evaluate the importance of nodes in the PPI network. The top 15 overlapping DEGs in the integrated results obtained with the two algorithms were considered significant genes.

### Verification of Hub Genes

We used the “sva” package of R software to eliminate the batch effect on the original expression of hub genes and the “ggplot” and “ggpubr” packages to analyze the normalized expression matrix and generate a violin plot.

ROC curve analysis was performed using the “pROC” package of R software to evaluate the diagnostic accuracy of each hub gene, and the sensitivity and specificity were assessed based on area under the ROC curve (AUC). Hub genes with the top 5 AUC values were selected as key genes.

### Constructing Coexpression Networks (Modules) for IgAN Samples

The original GSE93798 dataset included 20 IgAN patients (Supplementary Table 1), 22 healthy controls, and their clinical data and was deemed suitable for WGCNA. However, the investigators who generated the original dataset considered that only 19 samples met the research requirements of their study. We found that the age and sex of two samples derived from IgAN patients were the same, creating a mismatch between the clinical data and sample number; we therefore discarded these two samples and ultimately included 17 IgAN samples in our WGCNA.

The gene expression matrix of the 17 IgAN samples from GSE93798 was constructed, and the top 25% of genes with the largest variance in the samples were selected for WGCNA using the WGCNA package of R v4.0.0 software. Outlier samples were detected by hierarchical clustering, and an appropriate soft threshold power was selected in order to achieve standard scale-free networks. The next stage involved the construction of an adjacency matrix and topologic overlap matrix (TOM) and the calculation of corresponding dissimilarity (1 – TOM); a gene dendrogram was generated and module identification was performed by dynamic tree cutting with the minimum module size set to 30. The clustering of module eigengenes was carried out to merge highly similar modules with a dissimilarity of 0.25. The gene network was constructed with 1,000 randomly selected genes.

### Analysis of the Relationship Between Coexpression Modules and Clinical Features of IgAN

Module eigengenes were defined as the first principal component of the expression matrix for a given module and represented the average gene expression level for all genes in each module. Demographic and clinical information including sex, age, disease stage, and renal function were converted to numerical values, and regression analysis was performed between these and the module eigengene values. MM was defined as the association between a gene and a given module, and GS was defined as the correlation between genes and clinical features. Genes with high GS and MM for a clinical feature were retained as candidates for subsequent analysis. All analyses were performed using the WGCNA package of R v4.0.0 software.

### Functional Enrichment Analysis of Coexpression Modules

We used the Database for Annotation, Visualization and Integrated Discovery (DAVID; http://david.abcc.ncifcrf.gov/) (Huang da et al., 2009) to perform Gene Ontology (GO) function and Kyoto Encyclopedia of Genes and Genomes (KEGG) pathway enrichment analyses for overlapping DEGs, with *P* < 0.05 and gene count ≥2 used as cutoffs. The modules of interest were visualized using STRING (string-db.org/cgi/input.pl).

### Reagents

Antibodies against periostin (POSTN; sc-398631) and glyceraldehyde 3-phosphate dehydrogenase (sc-66163) were purchased from Santa Cruz Biotechnology (Santa Cruz, CA, USA). Recombinant mouse transforming growth factor β1 (TGF-β1) (P04202) was from MedChemExpress (Princeton, NJ, USA). Antibodies against proliferating cell nuclear antigen (PCNA; #13110) and B cell lymphoma-associated X protein (BAX; #2772) were from Cell Signaling Technology (Danvers, MA, USA). Recombinant mouse POSTN/osteoblast-specific factor (OSF)-2 protein (2955-F2-050) was from R&D Systems (Minneapolis, MN, USA). Cell Counting Kit 8 (CCK-8) was from Dojindo (Shanghai, China). The terminal deoxynucleotidyl transferase dUTP nick end labeling (TUNEL) assay kit was from Roche (Basel, Switzerland). Horseradish peroxidase-conjugated goat anti-mouse IgG H&L (ab6789) and goat anti-rabbit IgG H&L (ab205718) were from Abcam (Cambridge, MA, USA). Small interfering (si)RNA against *POSTN* and a scrambled negative control (NC) siRNA were obtained from Genomeditech (Shanghai, China) and had the following sequences: *POSTN*, 5′-CAAUGUCAAUGUUGAGCUA UAGCUCAACAUUGACAUUG-3′ and scrambled, 5′-UUCUCCGAACGUGUCACGUdTdT ACGUGACACGUUCGGAGAAdTdT-3′. *POSTN* RNA primers were synthesized by Sangon Biotech (Shanghai, China) and had the following sequences: forward, 5′-AAGGGAATGACTAGCGAAGAAA-3′ and reverse, 5′-CTCATTCACTAGAAGCGTTTCG-3′.

### *In vitro* Experiments Using MMCs

MMCs were obtained from the Cell Bank of the Chinese Academy of Sciences (Shanghai, China) and cultured in Dulbecco's modified Eagle's medium-F12 with 10% fetal bovine serum. After serum starvation for 24 h, the cells were stimulated with different concentrations (1, 2.5, 5, 10, and 20 ng/ml) of TGF-β1 for 48 h and recombinant mouse POSTN protein for 24 h. siRNA against POSTN was used to knock down *POSTN* gene expression in MMCs, which were then stimulated with TGF-β1 (20 ng/ml) for 48 h.

Western blotting and real-time (RT)-PCR were used to detect protein and mRNA levels of POSTN, respectively. BAX and PCNA levels were also evaluated by western blotting. Cell proliferation was assessed using CCK-8, and apoptotic cells were detected with the TUNEL assay.

### Collection of Clinical Specimens

We enrolled 120 inpatients who underwent renal biopsy at the Nephrology Department of our hospital from June 2019 to June 2020 after the patients provided informed consent to participate in the study. We collected clinical data and renal biopsy tissue specimens from 41 patients diagnosed with IgAN. The exclusion criteria were as follows: patients with diabetes, hepatitis, cirrhosis, severe metabolic syndrome, systemic lupus erythematosus, and secondary IgAN. According to these criteria, five patients with hyperlipidemia and one with hepatitis B were excluded; therefore, 35 patients were ultimately included in the study (Supplementary Table 2). Additionally, 15 patients (Supplementary Table 3) with kidney cancer were recruited at the Department of Urology of our hospital, and kidney tissue samples were obtained from a site 5 cm beside the cancer in these patients and used as control tissue.

### Immunohistochemistry and TUNEL of Glomeruli in Renal Tissue

Immunohistochemistry (IHC) was performed to analyze POSTN and PCNA expression in paraffin-embedded renal tissue sections. TUNEL was used to detect apoptotic glomerular mesangial cells. Two pathologists blinded to group assignment counted cells in five randomly selected fields in areas of positive staining. A semiquantitative analysis was performed using Image-Pro Plus v6.0 software (Media Cybernetics, Rockville, MD, USA) to assess protein levels of hub genes in the glomerulus region based on average optical density measurements (integrated optical density/glomeruli area).

### Statistical Analysis

Prism 7 software (GraphPad, La Jolla, CA, USA) was used for data analysis. Data are expressed as mean ± SEM. Differences between means were evaluated by analysis of variance and the two-tailed unpaired Student's *t*-test. *P* < 0.05 was considered statistically significant.

## Results

### Identification and GO Analysis of Upregulated Overlapping DEGs

**Figure 2:**
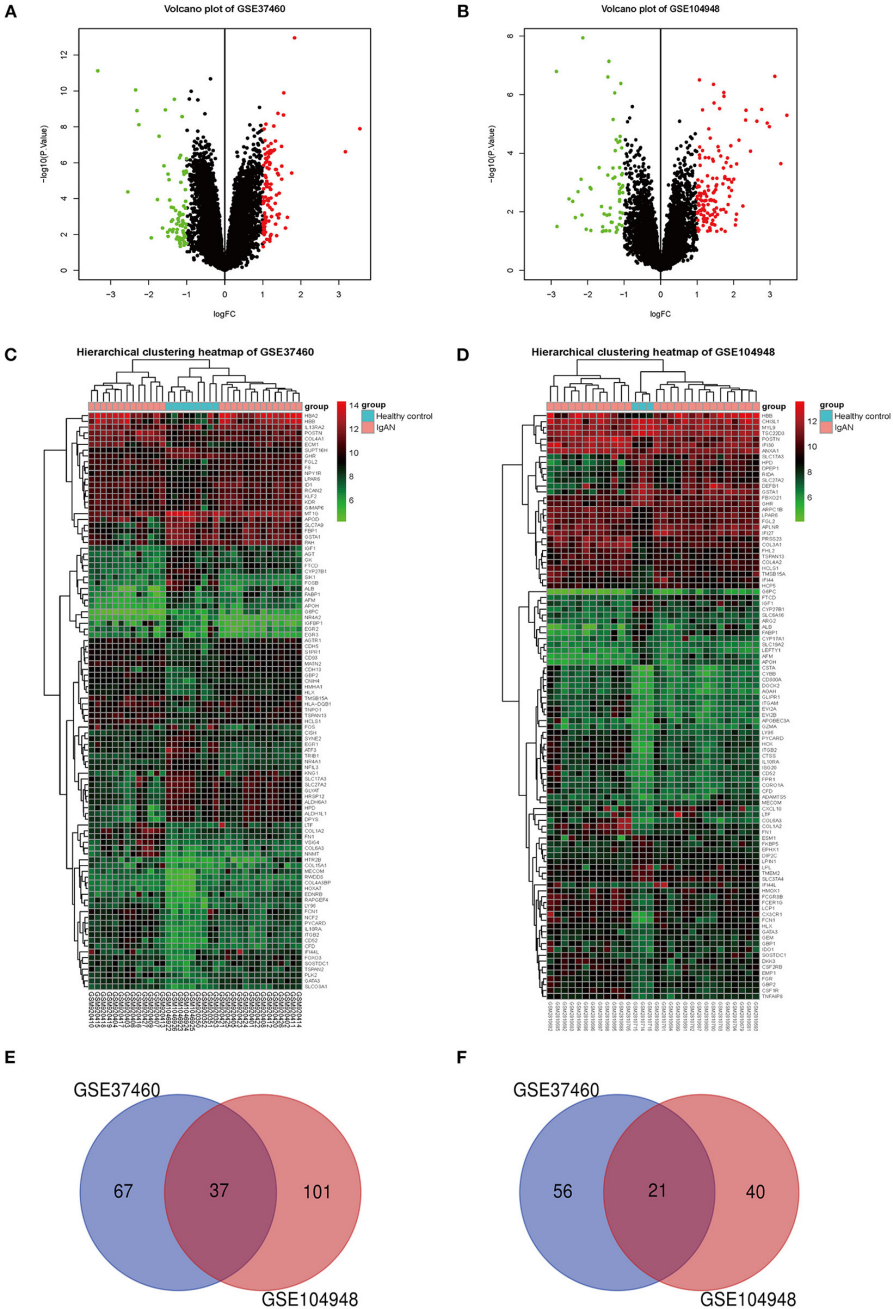
DEG screening and identification of overlapping DEGs. **(A,B)** Volcano plot of DEGs in the GSE37460 (*n* = 181) **(A)** and GSE104948 (*n* = 199) **(B)** datasets. Red and green points represent upregulated and downregulated genes, respectively, based on |fold change (FC)| ≥1.0 and adjusted *P* < 0.05. The black points represent genes with no significant difference. **(C,D)** Hierarchical clustering heatmap of the top 100 DEGs of the GSE37460 **(C)** and GSE104948 **(D)** datasets based on |FC| ≥1.0 and adjusted *P* < 0.05. The Venn diagram shows overlapping DEGs of GSE37460 and GSE104948. **(E,F)** The 37 upregulated **(E)** and 21 downregulated **(F)** overlapping DEGs in the two datasets.

After normalization of GSE37460 and GSE104948 datasets (Supplementary Figure 1), we performed a screen of DEGs using the R software. In the GSE37460 dataset, we identified 181 DEGs based on the cutoff values, including 104 genes that were upregulated and 77 that were downregulated in IgAN samples relative to healthy controls (Supplementary Table 4). In the GSE104948 dataset, there were 199 DEGs, including 138 that were upregulated and 61 that were downregulated compared with the control (Supplementary Table 5). All DEGs and hierarchical cluster (heatmap) analysis of the top 100 DEGs in the two datasets are shown in Figures 2A–D. There were 37 upregulated and 21 downregulated DEGs that overlapped between the GSE37460 and GSE104948 datasets (Figures 2E,F and Table 2). GO and KEGG analyses of the 37 upregulated overlapping DEGs are shown in Supplementary Figure 2 and Supplementary Tables 6, 7.

**Table 2:** Overlapping differentially expressed genes (DEGs) in the GSE93798 and GSE104948 datasets^*^.

**Direction of gene expression change**	**Overlapping DEGs**
Upregulated (*n* = 37)	*IFI44L, GIMAP4, ACTA2, C1QA, IL10RA, FGL2, C8orf4, ITGB2, FCN1, LPAR6, NCF2, TYROBP, CD52, NETO2, GMFG, GATA3, TMSB15A, TGFBI, LTF, SOSTDC1, COL1A2, CD53, LY96, COL6A3, POSTN, CFD, PYCARD, PHLDA2, TSPAN13, GBP2, HLX, HBB, FN1, ISG15, HCLS1, MECOM, KIAA1462*
Downregulated (*n* = 21)	*UPB1, ALB, CXCL14, SLC22A8, GSTA1, IGF1, HPD, CYP27B1, FTCD, PRODH2, AFM, FABP1, PCK1, GHR, PBLD, UMOD, SLC27A2, SLC17A3, PAH, G6PC, APOH*

* DEGs were obtained using the limma package of R software according to a cutoff of |fold change| ≥1.0 and adjusted P < 0.05.

### Hub Gene Identification and Verification of Gene Expression and ROC Curve Analysis

**Figure 3:**
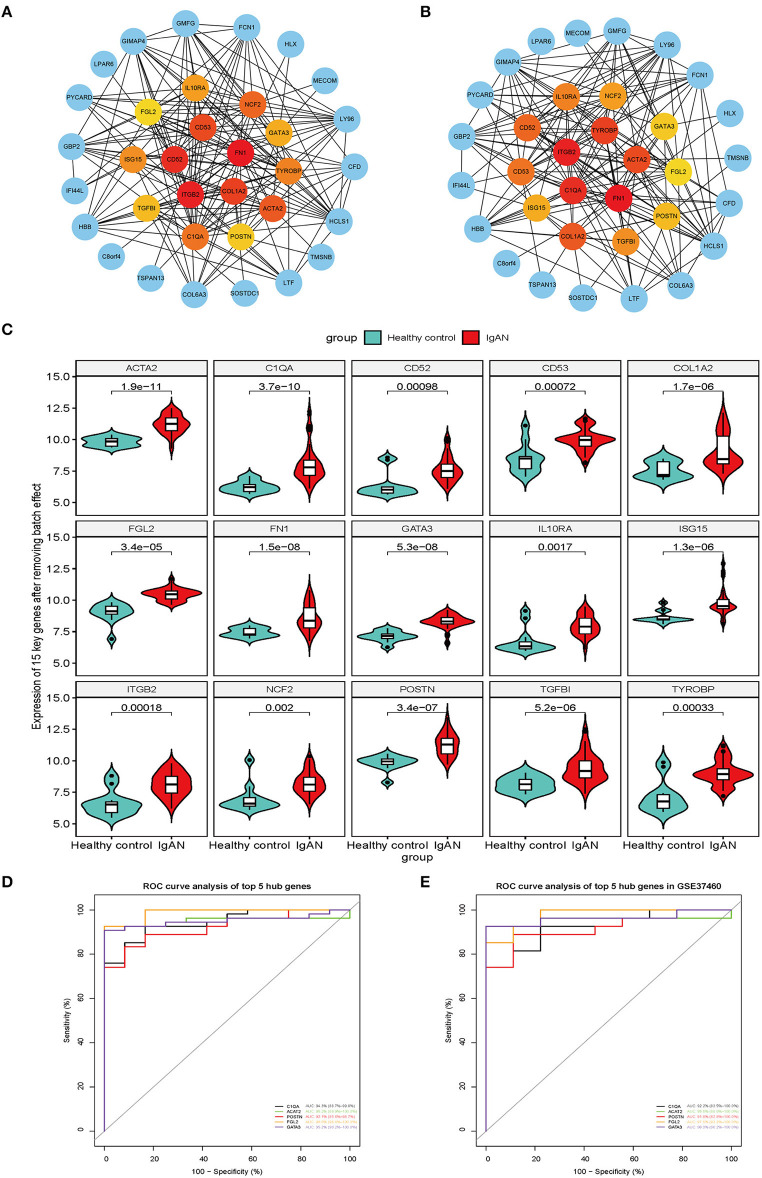
Screen of upregulated overlapping DEGs for key genes. **(A,B)** Top 15 upregulated overlapping DEGs analyzed with the stress **(A)** and betweenness **(B)** algorithms of cytoHubba plugin in Cytoscape, shown in yellow, orange, and red in the middle of the circle. Different colors represent different connectivity with other genes in the PPI network. **(C)** Violin plot of the gene expression analysis of the 15 key genes after eliminating the batch effect of the original analysis. The 15 genes were more highly expressed in IgAN patients than in healthy control subjects (*P* < 0.01). **(D)** Screen of the top five key genes based on AUC value in the ROC curve analysis. The ROC plot shows AUC (95% CI), sensitivity, and specificity values for *POSTN, GATA3, C1QA, ACAT2*, and *FGL2*. **(E)** Verification of the five hub genes (*POSTN, GATA3, C1QA, ACAT2*, and *FGL2*) in GSE37460 by ROC curve analysis, with AUC (95% CI), sensitivity, and specificity values.

The top 15 hub genes and sub-PPI modules were identified using cytoHubba (Figures 3A,B). We compared the hub genes and the top 15 DEGs identified using the stress and betweenness algorithms of cytoHubba (Supplementary Table 8) and found that they matched.

The results of the original gene expression analysis of the 15 hub genes were visualized using a violin plot (Figure 3C and Supplementary Table 9). We performed an ROC curve analysis and compared the AUC values of the 15 hub genes to evaluate their sensitivity and specificity for diagnosing IgAN. Five of the hub genes [*POSTN*, GATA-binding protein (*GATA3*), complement C1q A chain (*C1QA*), acetyl-coenzyme A acetyltransferase 2 (*ACAT*), and fibrinogen-like protein (*FGL2*)] had an AUC >0.90 and were retained as the genes with the highest potential diagnostic value for IgAN (Figure 3D and Table 3). To confirm their clinical utility, we performed an ROC curve analysis with the GSE37460 dataset; *POSTN* showed the highest AUC (91.8%; 95% CI: 82.8–100.0%) (Figure 3E).

**Table 3:** Receiver-operating characteristic curve analysis of five hub genes for IgAN.

**Hub genes**	**AUC**	**95% CI**	***P*-value**	**Youden index**	**Sensitivity (%)**	**Specificity (%)**
*ACAT2*	0.952	0.899–1.000	<0.0001	0.9259	92.59	100.00
*C1QA*	0.943	0.887–0.998	<0.0001	0.7685	85.19	91.67
*POSTN*	0.921	0.856–0.987	<0.0001	0.7500	83.33	91.67
*GATA3*	0.952	0.902–1.000	<0.0001	0.9074	90.74	100.00
*FGL2*	0.988	0.968–1.000	<0.0001	0.9259	92.59	100.00

AUC, area under the receiver-operating characteristic curve; CI, confidence interval; IgAN, immunoglobulin A nephropathy.

### WGCNA Construction and Identification of Key Modules

WGCNA is a method for analyzing the expression patterns of multiple genes in different samples; genes with similar expression patterns are grouped into clusters or modules (Beckerman et al., 2017) and likely have the same biological functions. This allows the study of associations between modules and sample characteristics such as clinical features (Bao et al., 2020).

**Figure 4:**
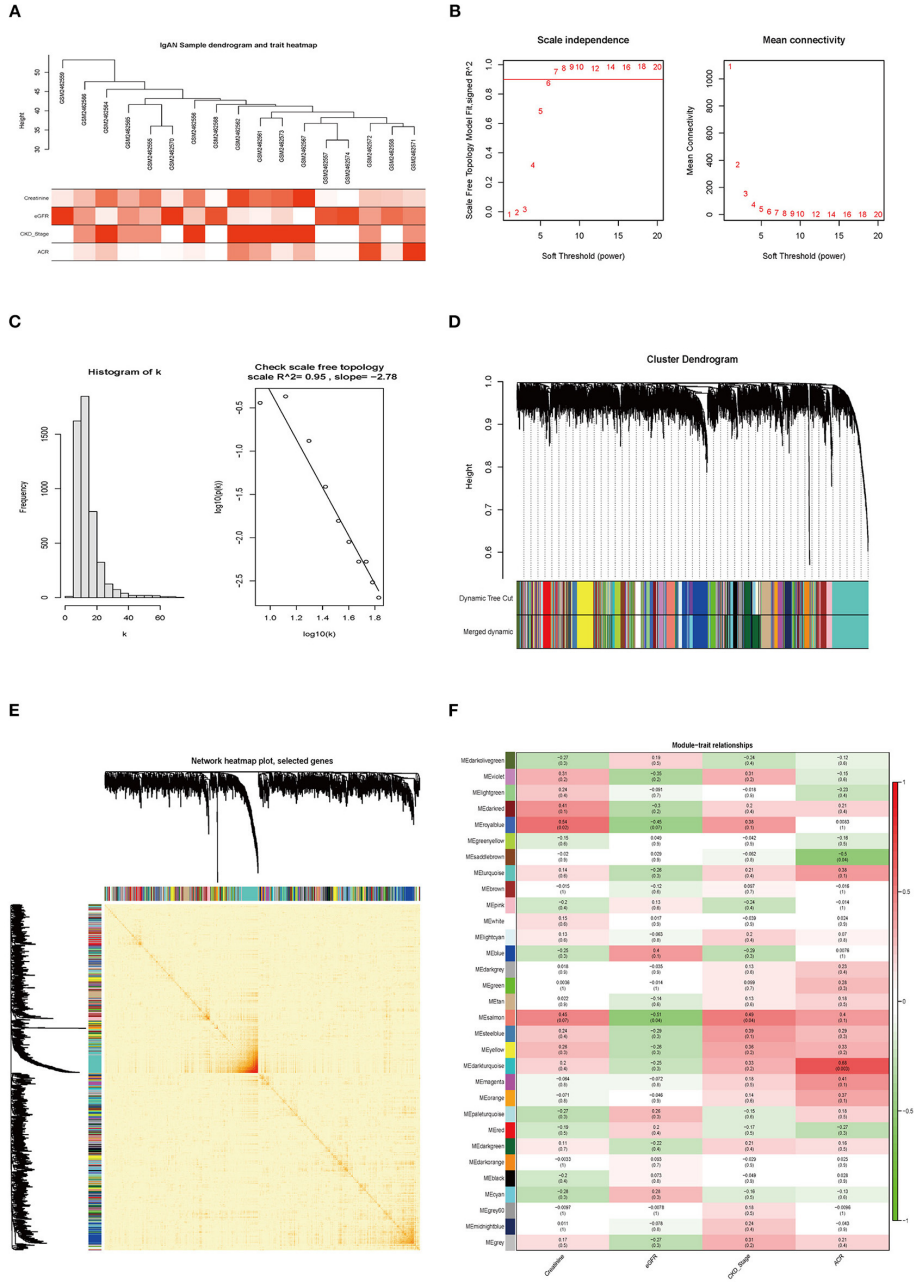
Weighted coexpression network construction and identification of the key module. **(A)** Hierarchical clustering dendrogram of samples from GSE93798. Clinical features of renal function are shown at the bottom. **(B,C)** Analysis of the scale-free fit index and mean connectivity for various soft-thresholding powers. The scale-free topology was tested for β = 7. **(D)** Hierarchical clustering dendrogram of dissimilar genes based on topologic overlap. Modules are the branches of the clustering tree. **(E)** Heatmap of TOM of 1,000 selected genes in WGCNA; darker and lighter colors represent higher and lower degrees of overlap, respectively. The gene dendrogram and module assignment are shown along the left side and top. **(F)** Correlation between module eigengenes and clinical features of IgAN. Each row corresponds to a module eigengene and columns represent clinical features. Each cell contains the correlation and *P*-value. The royalblue module was closely related to indices of kidney function (creatinine and eGFR).

A total of 17 samples with clinical data were included in the WGCNA (Figure 4A). We found 4,926 genes with altered expression in IgAN relative to control samples, and the top 25% were retained for further analysis. We selected β = 7 (scale-free *R*^2^ = 0.95) as the soft-thresholding power to obtain a scale-free network (Figures 4B,C). We identified 30 gene coexpression modules after excluding the gray module using a merged dynamic tree cut (Figure 4D). The heatmap of the TOM of 1,000 selected genes revealed that each module independently validated the others (Figure 4E). We also found that the module containing *POSTN* (i.e., the royalblue module) had the highest correlation with creatinine (*R*^2^ = 0.54, *P* = 0.02) and eGFR (*R*^2^ = −0.45, *P* = 0.07) (Figure 4F).

**Figure 5:**
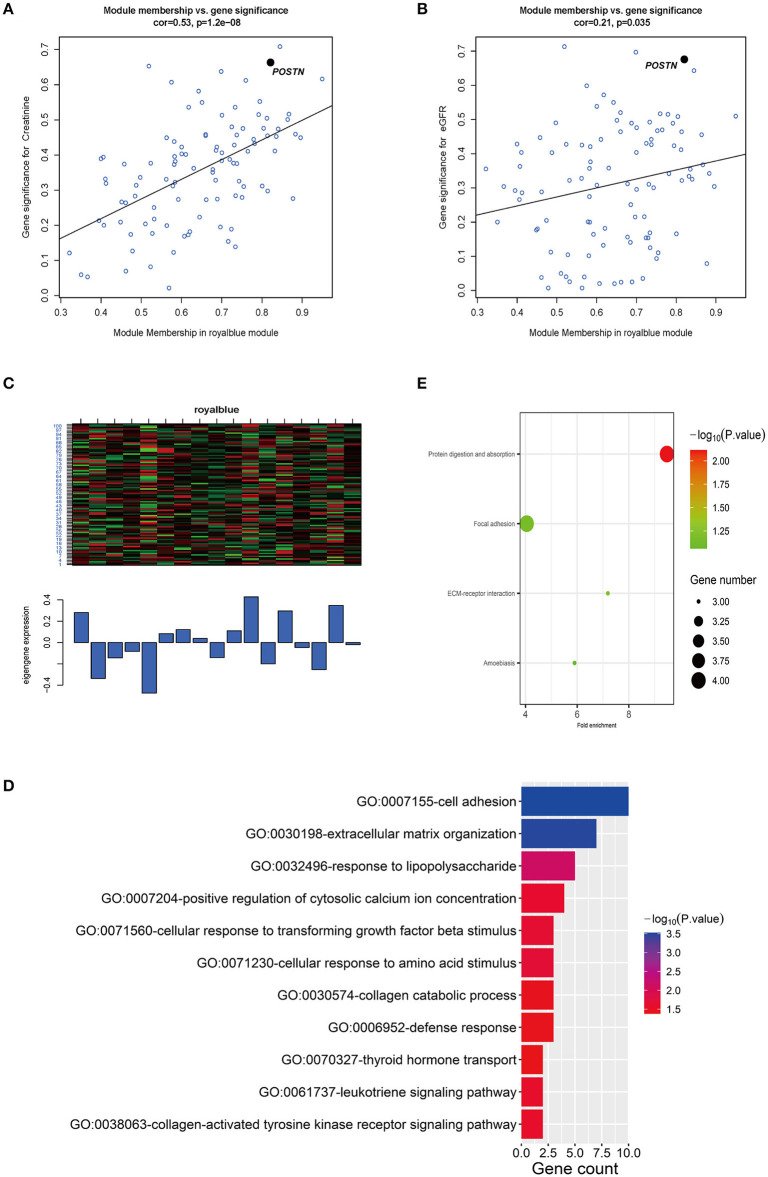
Relationship between key modules and clinical features and GO analysis of genes in the key module. **(A,B)** Scatterplots of GS vs. MM in the royalblue module with creatinine and eGFR of IgAN patients. The correlation between GS and creatinine and eGFR was 0.53 (*P* = 1.2e−08) and 0.21 (*P* = 0.035), respectively. *POSTN* had the highest MM and GS for creatinine (MM = 0.82 and GS = 0.66) and eGFR (MM = 0.82 and GS = −0.67) of all genes in the royalblue module. **(C)** Heatmap and bar graph of gene expression distribution of 101 genes in the royalblue module. **(D)** GO analysis of genes in the royalblue module, with threshold count ≥2 and *P* < 0.05. **(E)** Only one KEGG pathway (protein digestion and absorption) was enriched for the royalblue module; three additional pathways (ECM–receptor interaction, focal adhesion, and amoebiasis) are shown.

We generated scatterplots of GS vs. MM in the royalblue module with clinical information of IgAN patients. The GS values for creatinine and eGFR showed correlations of 0.53 (*P* = 1.2e−08) and 0.21 (*P* = 0.035), respectively (Figures 5A,B). Meanwhile, *POSTN* had very high MM and GS for creatinine (MM = 0.82 and GS = 0.66) and eGFR (MM = 0.82 and GS = −0.67) (Supplementary Table 10). Thus, *POSTN* was deemed as a key gene in the royalblue module. A heatmap and bar graph of gene expression distribution in the royalblue module are shown in Figure 5C.

### GO Enrichment Analysis and PPI Visualization of Genes in the Royalblue Coexpression Module

We used the DAVID online database to perform GO and KEGG enrichment analyses of genes in the royalblue coexpression module. The genes were significantly enriched in the following biological process GO terms: cell adhesion, extracellular matrix organization, response to lipopolysaccharide, cellular response to transforming growth factor beta stimulus, and others with the threshold set to *P* < 0.05 and count ≥2 (Figure 5D and Table 4). There were four enrichment KEGG pathways (protein digestion and absorption, ECM–receptor interaction, focal adhesion, and amoebiasis) of these genes (Figure 5E and Table 5).

**Table 4:** Biological processes of genes in the royalblue module in the weighted gene correlation network analysis.

**ID**	**Description**	**Count**	*P*-value^*^	**Genes**
GO:0007155	Cell adhesion	10	2.98E−04	*VCAM1, CDH13, WISP1, CD33, FAP, POSTN, IL32, ADAM12, PCDH17, EMILIN1*
GO:0030198	Extracellular matrix organization	7	3.21E−04	*VCAM1, TTR, COL4A2, COL4A1, POSTN, COL5A2, EMILIN1*
GO:0032496	Response to lipopolysaccharide	5	0.007469291	*VCAM1, THBD, SLPI, TAC1, LIAS*
GO:0071230	Cellular response to amino acid stimulus	3	0.02060527	*COL4A1, PDGFD, COL5A2*
GO:0071560	Cellular response to transforming growth factor β stimulus	3	0.022281384	*COL4A2, POSTN, PDGFD*
GO:0007204	Positive regulation of cytosolic calcium ion concentration	4	0.025308179	*S1PR3, AGTR1, CYSLTR1, TAC1*
GO:0038063	Collagen-activated tyrosine kinase receptor signaling pathway	2	0.027901906	*COL4A2, COL4A1*
GO:0061737	Leukotriene signaling pathway	2	0.027901906	*RGS1, CYSLTR1*
GO:0030574	Collagen catabolic process	3	0.036513389	*COL4A2, COL4A1, COL5A2*
GO:0006952	Defense response	3	0.039687023	*CYSLTR1, GATA3, IL32*
GO:0070327	Thyroid hormone transport	2	0.041563188	*TTR, SLCO4A1*

* Significantly enriched Gene Ontology terms of differentially expressed genes were determined according to the cutoff values of P <0.05 and count ≥2.

**Table 5:** Kyoto Encyclopedia of Genes and Genomes (KEGG) pathways of genes in the royalblue module in the weighted gene correlation network analysis.

**ID**	**Description**	**Count**	**%**	***P*-value**
hsa04974	Protein digestion and absorption	4	3.96039604	0.007676167
hsa04512	ECM–receptor interaction	3	2.97029703	0.061404602
hsa04510	Focal adhesion	4	3.96039604	0.069535569
hsa05146	Amoebiasis	3	2.97029703	0.086549375

The four enriched KEGG pathways of differentially expressed genes were displayed.

ECM, extracellular matrix.

**Figure 6:**
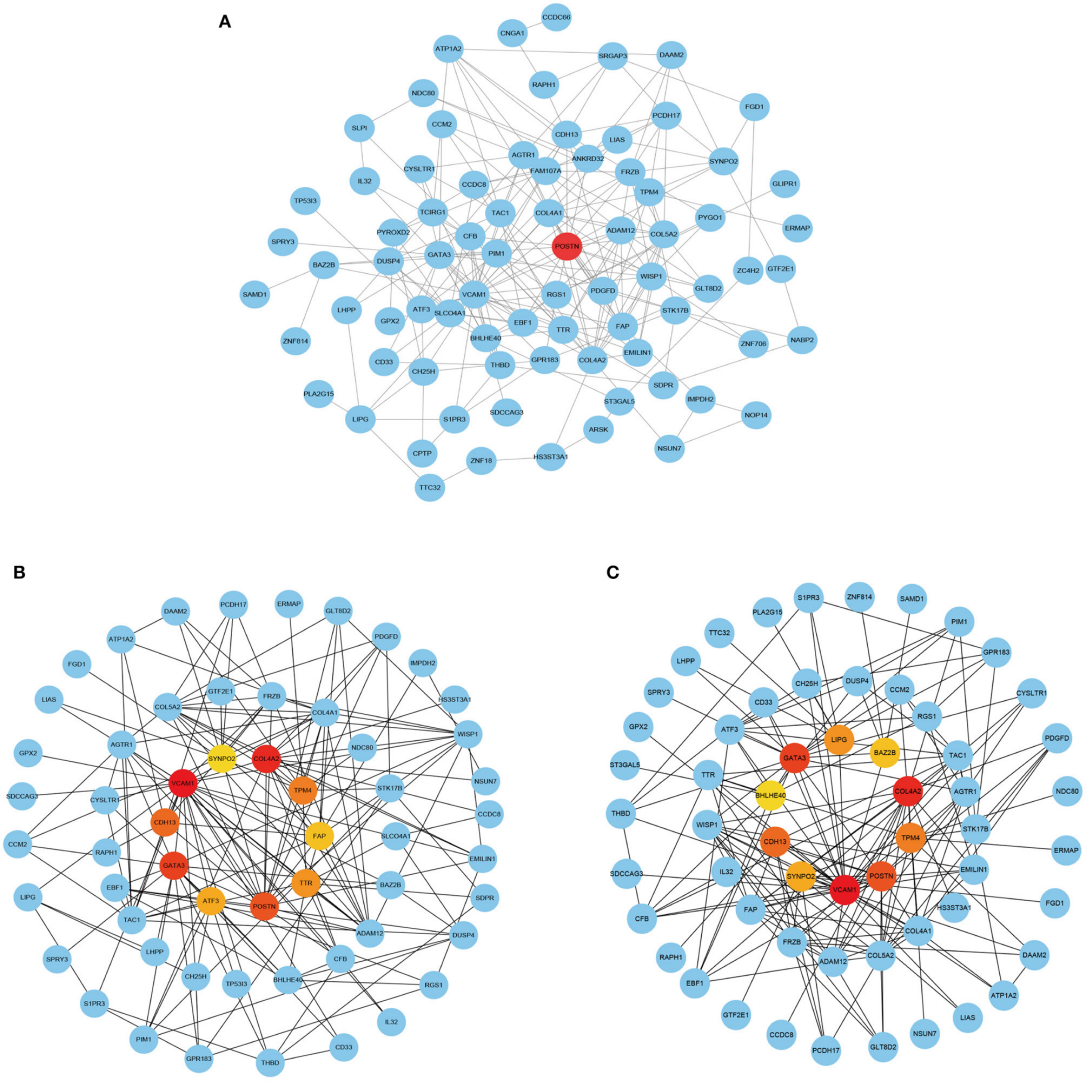
PPI network analysis of POSTN in the royalblue module. **(A)** PPI network analysis of genes in the royalblue module. **(B,C)** Top 15 genes in the royalblue module identified by the stress **(B)** and betweenness **(C)** algorithms in cytoHubba.

The PPI analysis of the genes in this module revealed POSTN at the core of the PPI network (Figure 6A). The stress (Figure 6B) and betweenness (Figure 6C) algorithms in cytoHubba confirmed that *POSTN* had high connectivity with other genes in the module.

### Effect of POSTN Overexpression on MMC Proliferation and Apoptosis

**Figure 7:**
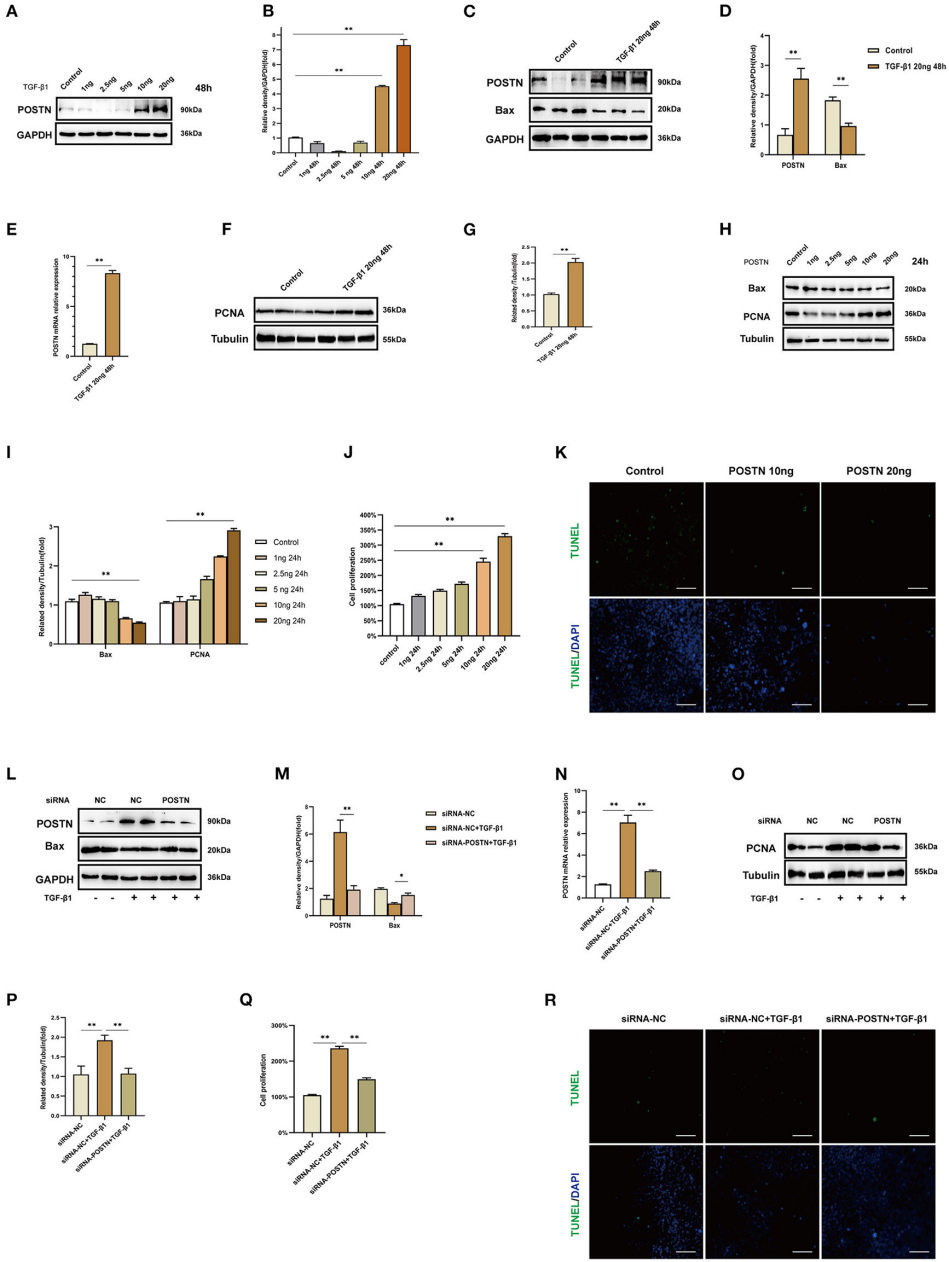
High levels of POSTN enhance the proliferation and suppress apoptosis in MMCs. **(A,B)** Immunoblot analysis and quantification of POSTN in MMCs treated with indicated concentrations of TGF-β1 for 48 h. **(C–I)** Immunoblot analysis and quantification of POSTN, BAX, and PCNA in MMCs treated with 20 ng/ml TGF-β1. **(H,I)** Immunoblot analysis and quantification of BAX and PCNA in MMCs treated with indicated concentrations of recombinant POSTN protein for 24 h. **(J)** Proliferation of MMCs as detected with the CCK-8 assay. **(K)** Detection of apoptotic cells with the TUNEL assay. Scale bar: 50 μm. **(L,M)** siRNA-mediated *POSTN* knockdown in MMCs. Immunoblot analysis and quantification of POSTN and BAX levels in TGF-β1–treated MMCs after *POSTN* knockdown. **(N)** *POSTN* mRNA level in TGF-β1–treated MMCs after *POSTN* knockdown. **(O,P)** Immunoblot analysis and quantification of PCNA in TGF-β1–treated MMCs after *POSTN* knockdown. **(Q,R)** Detection of proliferation and apoptosis with the CCK-8 and TUNEL assays, respectively, in TGF-β1–treated MMCs after *POSTN* knockdown. Scale bar: 50 μm. Data are presented as mean ± SEM (*n* = 3). *P < 0.05, **P < 0.01.

To validate the results of the WGCNA, we carried out *in vitro* experiments using MMCs. Exposure to different concentrations (1, 2.5, 5, 10, and 20 ng/ml) of TGF-β1 for 48 h significantly increased the level of POSTN (Figures 7A,B), with the highest expression observed at 20 ng/ml. Therefore, this concentration was used to stimulate MMCs in subsequent experiments.

Stimulation with TGF-β1 (20 ng/ml) for 48 h significantly increased POSTN and PCNA and decreased BAX expression (Figures 7C–G). Treatment of MMCs with different concentrations (1, 2.5, 5, 10, and 20 ng/ml) of recombinant POSTN protein for 24 h had the same effects on PCNA and BAX levels as TGF-β1 (Figures 7H,I). The results of the CCK-8 assay showed that cell proliferation was increased in a concentration-dependent manner upon exposure to recombinant POSTN; the rate of proliferation was the highest in cells treated with 20 ng POSTN (Figure 7J), whereas at this concentration, the rate of apoptosis was decreased (Figure 7K).

We used *POSTN* siRNA to silence *POSTN* in MMCs for 24 h, followed by stimulation with TGF-β1 (20 ng/ml) for 48 h. POSTN depletion reduced POSTN and PCNA levels and increased that of BAX (Figures 7L–P). Cell proliferation was also decreased upon siRNA-mediated knockdown of *POSTN* (Figure 7Q), whereas apoptosis was increased relative to cells transfected with NC siRNA and treated with TGF-β1 (Figure 7R).

### POSTN Expression in IgAN Patients

**Figure 8:**
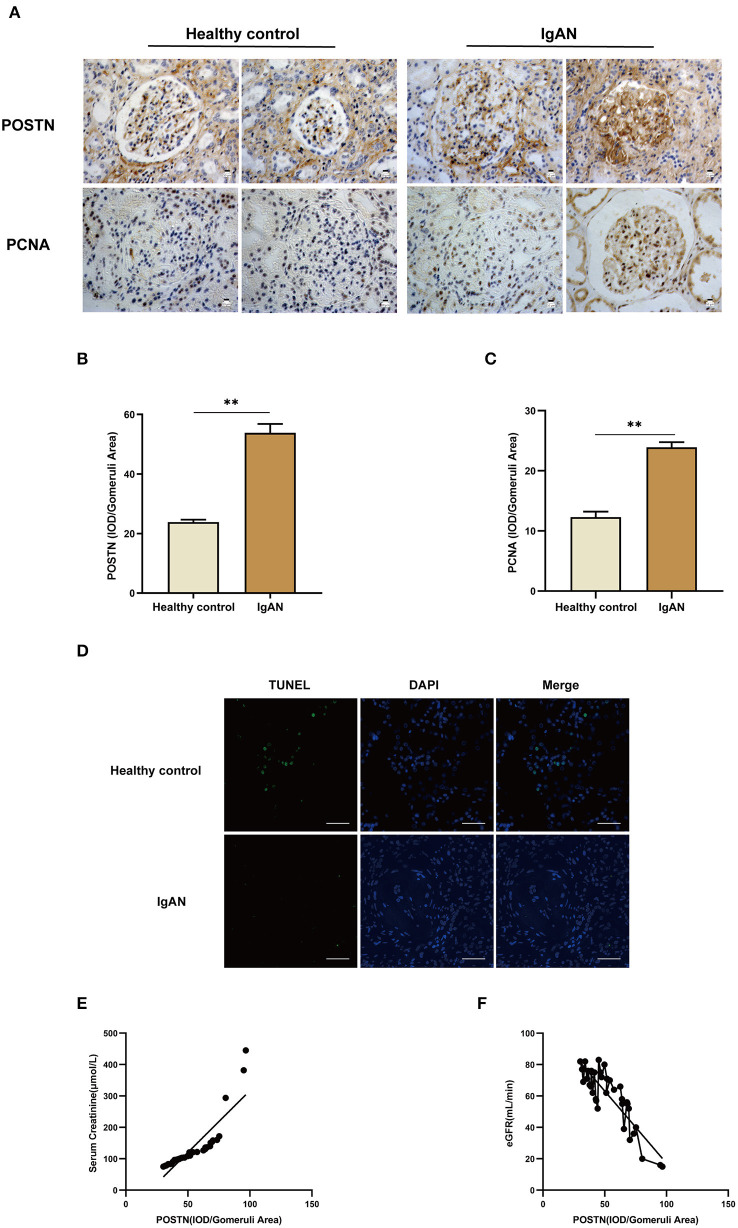
*POSTN* is highly expressed in the kidney of IgAN patients and is closely related to renal dysfunction. **(A–C)** IHC and quantification of POSTN and PCNA in human renal tissue. **(D)** TUNEL staining of renal tissue samples from IgAN patients and healthy controls. Scale bar: 50 μm. **(E,F)** Pearson correlation analysis of the correlation between POSTN and creatinine/eGFR. Data are presented as mean ± SEM (IgAN patients, *n* = 35 and healthy controls, *n* = 15). **P < 0.01.

We validated the clinical relevance of our findings in MMCs by IHC using renal tissue samples from IgAN patients and healthy control subjects (Table 6). POSTN and PCNA levels were increased, whereas the rate of apoptosis detected by TUNEL was decreased in IgAN patient samples compared with those of healthy subjects (Figures 8A–D). Moreover, POSTN level in the renal tissues of IgAN patients was positively correlated with serum creatinine level (*r* = 0.8603, *R*^2^ = 0.7401, 95% CI: 0.7388–0.9276) and negatively correlated with eGFR [*r* = −0.8673, *R*^2^ = 0.7522, 95% CI: (−0.9314)–(−0.7511)], suggesting that renal dysfunction in IgAN involves POSTN (Figures 8E,F).

**Table 6:** Demographic and clinical data of IgAN patients and healthy control subjects.

	**Healthy control**	**IgAN**
Sex
Male	9	20
Female	6	15
Age	58.1 ± 7.01	47.1 ± 10.5
Creatinine	50.9 ± 10.9	135.5 ± 80.4
Estimated glomerular filtration rate	109.1 ± 13.2	59.6 ± 18.7
Blood urea nitrogen	4.1 ± 0.6	6.7 ± 3.7
Cystatin C	0.67 ± 0/18	1.47 ± 0.73
Uric acid	324.9 ± 57.1	377.1 ± 90.2
24-h urine protein	136.9 ± 10.2	2,166.7 ± 1,682.4
Albumin-to-creatinine ratio	22.4 ± 3.5	887.8 ± 672.1

IgAN, immunoglobulin A nephropathy.

## Discussion

In this study, we investigated the relationship between key genes and clinical features of IgAN by *in silico* analyses of the GSE37460, GSE104948, and GSE93798 datasets, which were derived by high-throughput sequencing of IgAN patient kidney tissues. Although these datasets have been analyzed in previous studies, we validated the results of our analyses by performing *in vitro* experiments using MMCs and examining clinical specimens from IgAN patients. There were 37 upregulated DEGs that showed overlap between the GSE37460 and GSE104948 datasets (Figure 2E); we used the cytoHubba plugin of Cytoscape and ROC curve analysis to identify five key hub genes (Figures 3A–C), namely *POSTN, GATA3, C1QA, ACAT2*, and *FGL2* (Figures 3D,E and Table 3). GATA3 is a transcription factor of the GATA family (Labastie et al., 1994) that plays an important role in many cellular processes such as proliferation, development, and differentiation in various nonhematopoietic cells (Tong et al., 2000). C1QA triggers the activation of the classic complement pathway by binding immunoglobulin Fc in immune complexes and is involved in apoptosis (Korb and Ahearn, 1997). C1QA can act independently of the complement system as a cancer-promoting factor in the tumor microenvironment (Bulla et al., 2016). ACAT2, which is mainly expressed in the intestine and fetal liver, catalyzes the production of cholesteryl ester from cholesterol and long chain fatty acyl-CoA, thus providing cholesteryl esters for lipoprotein assembly (Anderson et al., 1998; Bemlih et al., 2010). Malignant progression of colorectal cancer has been linked to the pro-proliferative activity of ACAT2 (Weng et al., 2020). FGL2 is a membrane-bound or secreted protein expressed by macrophages, T cells, and tumor cells that has coagulation activity and immunosuppressive functions (Marazzi et al., 1998); it was found to promote mammary tumor progression by enhancing tumor angiogenesis or inducing epithelial-to-mesenchymal transition (Rabizadeh et al., 2015). POSTN was shown to induce proliferation in lung and gastric cancer cell lines (Hong et al., 2010; Kikuchi et al., 2014).

We used the GSE93798 dataset to perform WGCNA and identified a key module (royalblue) associated with renal function; within this module, the *POSTN* gene was the most highly correlated with kidney function indices (creatinine and eGFR). POSTN, also known as OSF-2, is a 90-kDa extracellular protein expressed during embryonic development and very early in postnatal tissue that was first identified in MC3T3-E1 osteoblastic cells (Takeshita et al., 1993; Kudo, 2011). POSTN overexpression promotes proliferation and migration in A549 lung cancer cells (Hong et al., 2010) and enhances proliferation in gastric cancer cell lines (Kikuchi et al., 2014), and recombinant POSTN and POSTN derived from normal human dermis stimulate melanoma cell proliferation (Kotobuki et al., 2014). POSTN is also known to play a role in the resistance of cancer cells to hypoxia-induced apoptosis (Bao et al., 2004). In the present study, treatment of MMCs with recombinant POSTN protein increased cell proliferation (Figures 7H,J).

IgAN has variable appearances in the kidney biopsy, ranging (by light microscopy) from normal to variable degrees of mesangial cell proliferation to florid crescentic necrotizing glomerular lesion or advanced sclerosing appearances (Working Group of the International Ig A Nephropathy Network the Renal Pathology Society et al., 2009). These initial lesions included vascular sclerosis, crescents, mesangial hypercellularity, endocapillary hypercellularity, segmental glomerulosclerosis, and tubulointerstitial fibrosis (Lai et al., 2011). Mesangial hypercellularity is a key feature of renal pathology (Working Group of the International Ig A Nephropathy Network the Renal Pathology Society et al., 2009). A VALIGA study examined 1,147 patients from 13 European countries that encompassed the whole spectrum of IgAN. M, S, and T lesions independently predicted the loss of eGFR and a lower renal survival (Coppo et al., 2014). The above literature shows that mesangial hypercellularity is an independent poor prognostic indicator for IgAN. Thus, the pro-proliferative function of POSTN is associated with a poor outcome for IgAN.

There is limited information on the role of POSTN in kidney disease. One study showed that POSTN promotes cell proliferation and macrophage polarization to drive tissue repair after acute kidney injury (Kormann et al., 2020), and POSTN was found to contribute to renal and cardiac dysfunction in rats with chronic kidney disease (CKD) (Prakoura and Chatziantoniou, 2017; Bian et al., 2019b). Additionally, *POSTN* knockdown attenuated 5/6 nephrectomy-induced intrarenal activation of the renin–angiotensin system, fibrosis, and inflammation in rats, implying a role in the development of CKD (Bian et al., 2019a). In a risk prediction model of ovarian cancer, POSTN combined with two other markers had high prognostic accuracy (Riester et al., 2014), and another study found that polycystic ovary syndrome is associated with elevated POSTN levels (Chen et al., 2019). Although POSTN levels in human urine may have prognostic value in IgAN, this has not been confirmed (Hwang et al., 2016). A study of glomerular proteomics found that POSTN may be a marker of IgAN injury (Paunas et al., 2019); unfortunately, this finding lacks basic follow-up experiments and clinical studies to verify the data. As IgAN lesions mainly reflect glomerular damage, we focused on the expression of POSTN in glomeruli and the association with renal pathology. We observed that POSTN was highly expressed in the kidney tissue of patients with IgAN compared with healthy control subjects and was related to renal dysfunction (i.e., elevated creatinine and decreased eGFR). However, additional studies are needed in order to determine the molecular basis of these observations.

Our study has some shortcomings including the limited period of enrollment that restricted the sample size. However, we were nonetheless able to identify hub genes in the IgAN gene expression network that can serve as potential therapeutic targets. Based on the present findings, we intend to analyze the relationships between serum and urine POSTN levels in IgAN patients before renal biopsy, the level in kidney tissue after renal biopsy, and the development of IgAN.

## Conclusion

The results of this study demonstrate that upregulation of POSTN promotes renal dysfunction in IgAN by stimulating proliferation and inhibiting apoptosis in mesangial cells. Thus, blocking POSTN is a potential therapeutic strategy for preventing IgAN and its progression to end-stage kidney disease.

## Data Availability Statement

The datasets generated for this study can be found in online repositories. The names of the repository/repositories and accession number(s) can be found in the article/Supplementary Material.

## Ethics Statement

The studies involving human participants were reviewed and approved by Renji Hospital Ethics Committee of Shanghai Jiaotong University School of Medicine (approval number: KY[2019]002). The patients/participants provided their written informed consent to participate in this study.

## Author Contributions

ZN conceived the study and reviewed and revised the manuscript. JW and QL performed the dataset analysis and experiments, analyzed the experimental data, and drafted the manuscript. SL, XS, and XZ participated in clinical sample collection. WZ and MZ analyzed the results of the TUNEL assay and IHC. All authors contributed to the article and approved the submitted version.

## Conflict of Interest

The authors declare that the research was conducted in the absence of any commercial or financial relationships that could be construed as a potential conflict of interest.

## Supplementary Material

Supplementary Figure 1 Boxplot of original and normalized gene expression in GEO datasets. **(A,B)** Boxplot of original gene expression in GSE37460 **(A)** and GSE104948 **(B)**. **(C,D)** Boxplot of normalized gene expression in GSE37460 **(C)** and GSE104948 **(D)**.

Supplementary Figure 2 GO and KEGG pathway analyses of 37 upregulated genes overlapping between GSE37460 and GSE104948. **(A–C)** GO analysis of 37 genes showing Biological process **(A)**, Cellular component **(B)**, and Molecular function **(C)** categories with threshold count ≥2 and *P* < 0.05. **(D)** KEGG pathway analysis showing pathways associated with the 37 upregulated genes according to a threshold count ≥2 and *P* < 0.05.

Supplementary Figure 3 Relationship between key modules and clinical features of IgAN in the royalblue module. **(A,B)** Scatterplots of GS vs. MM in the royalblue module with albumin:creatinine ratio (ACR) and CKD stage in IgAN patients. Correlations between GS and creatinine and eGFR were – 0.34 (*P* = 5e−04) and 0.11 (*P* = 0.27), respectively.

Supplementary Figure 4 Cluster trees and heatmaps for creatinine and eGFR in different modules in the WGCNA. **(A)** Creatinine. **(B)** eGFR.

Supplementary Table 1 Clinical and demographic information of patients with IgAN in the GSE93798 dataset.

Supplementary Table 2 Clinical and demographic information of the 35 IgAN patients in this study.

Supplementary Table 3 Clinical and demographic information of the 15 healthy control subjects in this study.

Supplementary Table 4 DEGs in dataset GSE37460.

Supplementary Table 5 DEGs in dataset GSE104948.

Supplementary Table 6 Significant GO terms for 37 genes upregulated in both GSE37460 and GSE104948.

Supplementary Table 7 Significant KEGG pathways for the 37 genes upregulated in both GSE37460 and GSE104948.

Supplementary Table 8 Top 15 upregulated overlapping DEGs identified using the stress and betweenness algorithms in cytoHubba.

Supplementary Table 9 Expression of 15 hub genes after eliminating the batch effect.

Supplementary Table 10 GS and MM of genes and clinical features of IgAN in the royalblue module.
