# Supplementary figures and images for: Periostin Contributes to Immunoglobulin a Nephropathy by Promoting the Proliferation of Mesangial Cells: A Weighted Gene Correlation Network Analysis
Source: Front Genet. 2021 Jan 7;11:595757. doi: 10.3389/fgene.2020.595757 (PMC7817997; doi:10.3389/fgene.2020.595757)

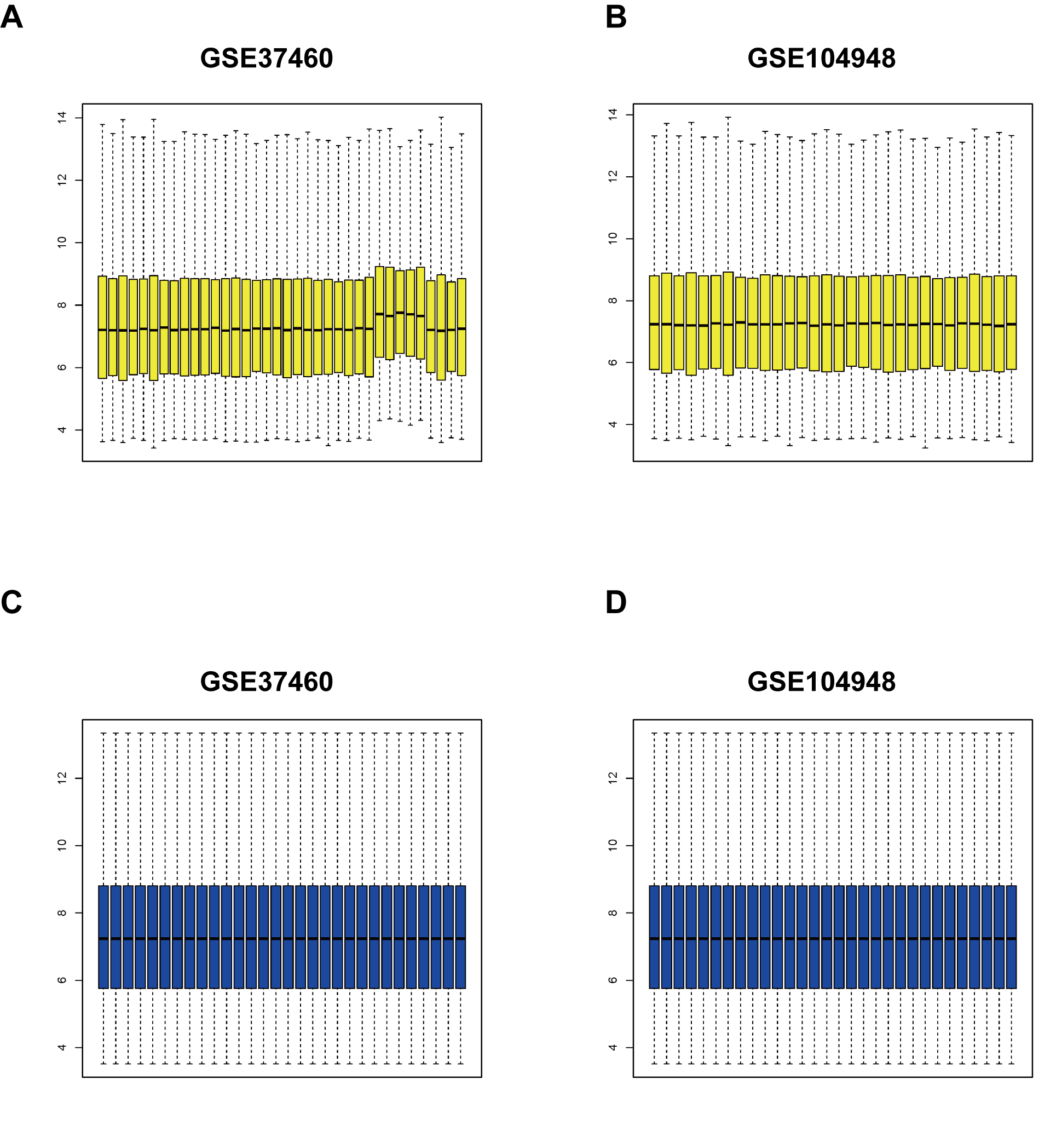

Supplement: Supplementary Figure 1 — Boxplot of original and normalized gene expression in GEO datasets. (A,B) Boxplot of original gene expression in GSE37460 (A) and GSE104948 (B). (C,D) Boxplot of normalized gene expression in GSE37460 (C) and GSE104948 (D). [file Image_1.TIF]

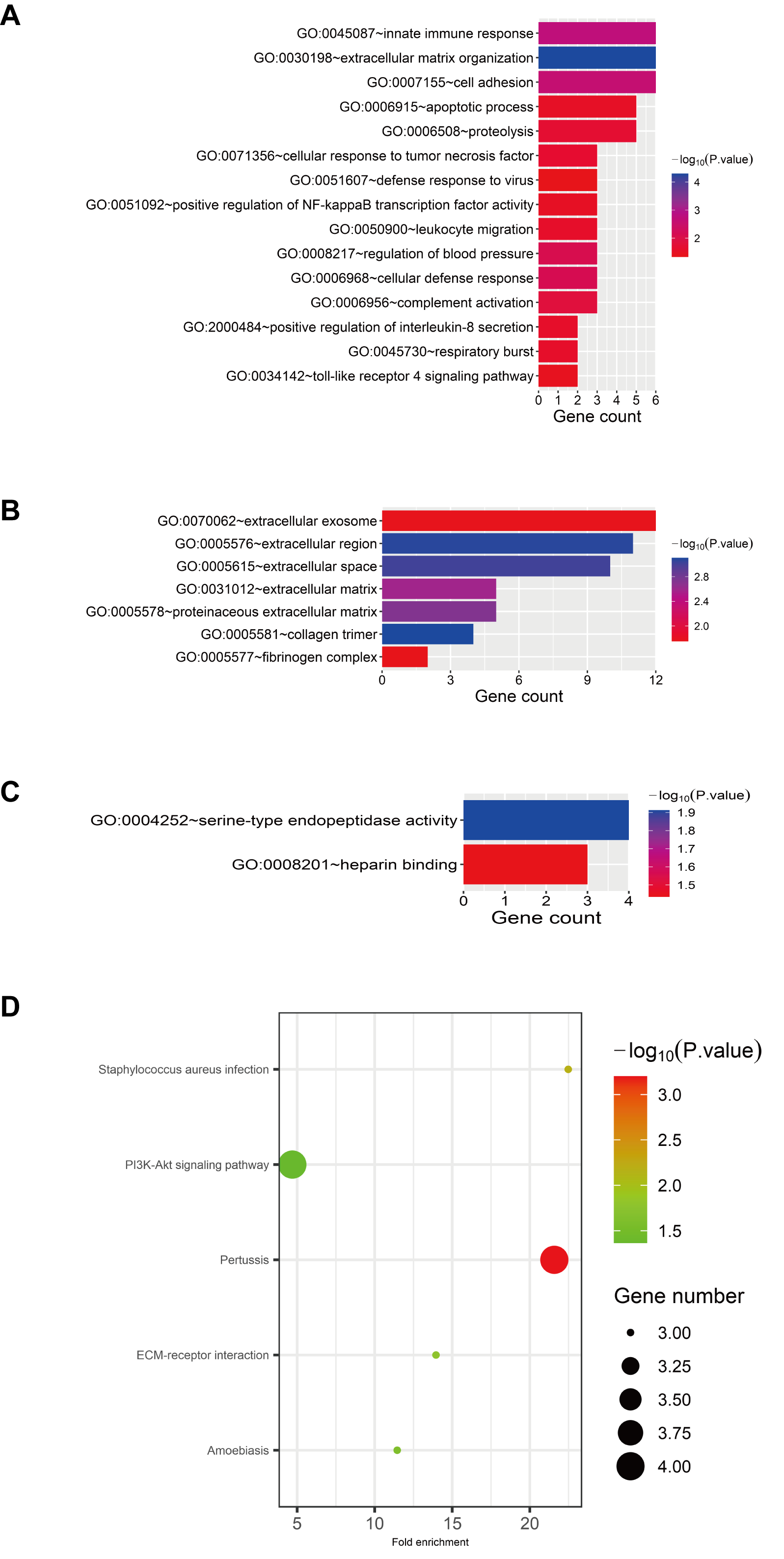

Supplement: Supplementary Figure 2 — GO and KEGG pathway analyses of 37 upregulated genes overlapping between GSE37460 and GSE104948. (A–C) GO analysis of 37 genes showing Biological process (A), Cellular component (B), and Molecular function (C) categories with threshold count ≥2 and P < 0.05. (D) KEGG pathway analysis showing pathways associated with the 37 upregulated genes according to a threshold count ≥2 and P < 0.05. [file Image_2.TIF]

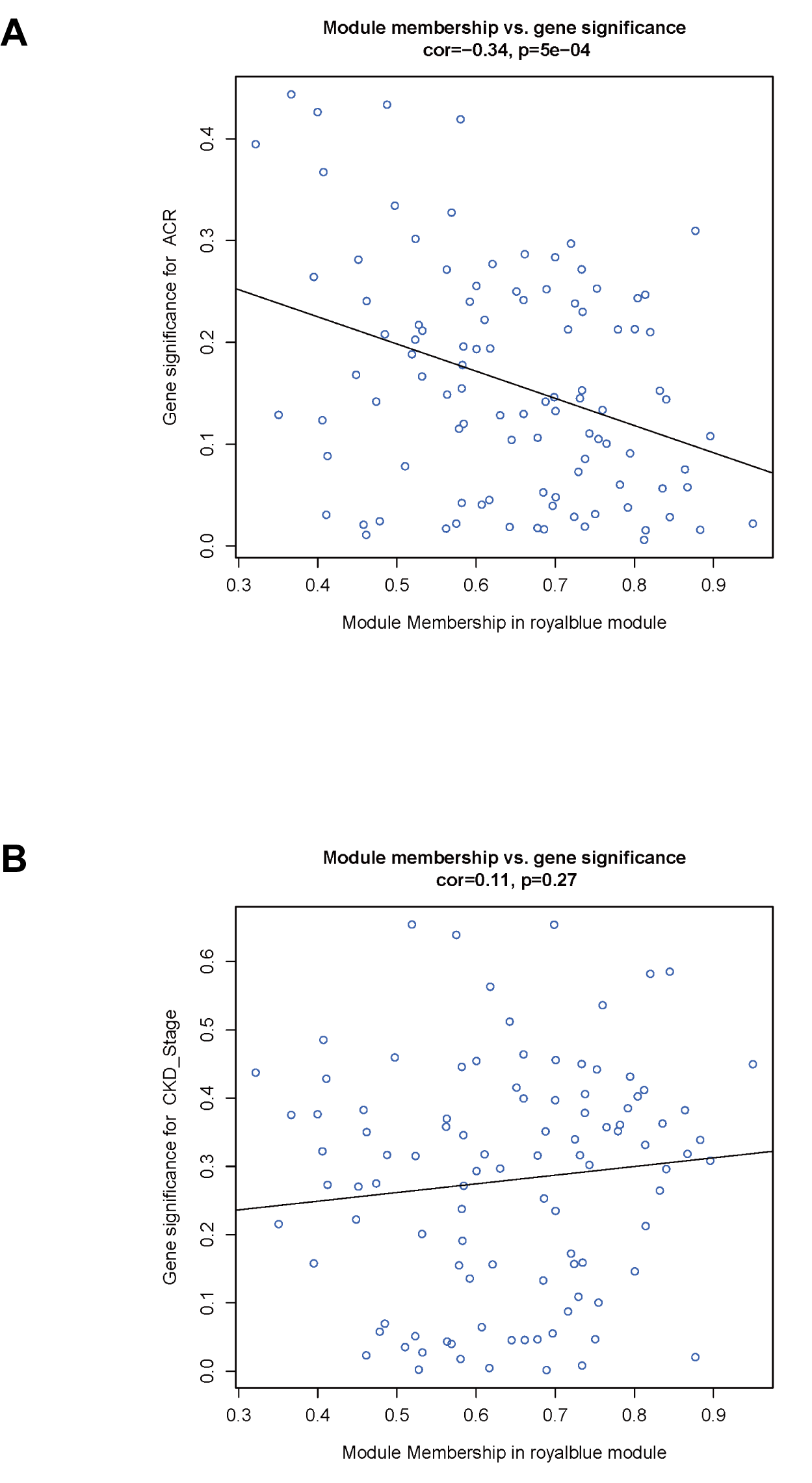

Supplement: Supplementary Figure 3 — Relationship between key modules and clinical features of IgAN in the royalblue module. (A,B) Scatterplots of GS vs. MM in the royalblue module with albumin:creatinine ratio (ACR) and CKD stage in IgAN patients. Correlations between GS and creatinine and eGFR were – 0.34 (P = 5e−04) and 0.11 (P = 0.27), respectively. [file Image_3.TIF]

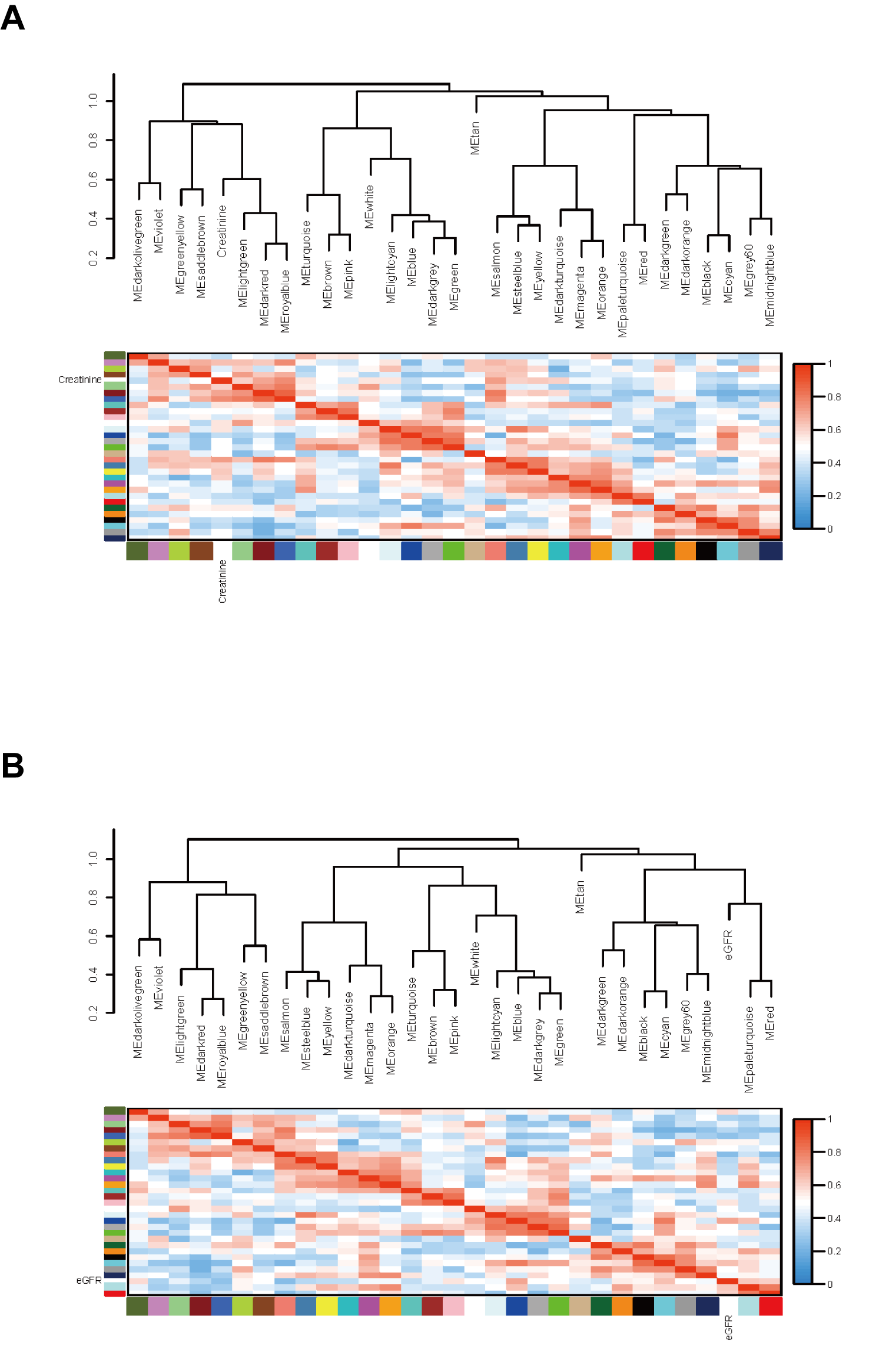

Supplement: Supplementary Figure 4 — Cluster trees and heatmaps for creatinine and eGFR in different modules in the WGCNA. (A) Creatinine. (B) eGFR. [file Image_4.TIF]
