# Supplementary material for: Periostin Contributes to Immunoglobulin a Nephropathy by Promoting the Proliferation of Mesangial Cells: A Weighted Gene Correlation Network Analysis
Source: Front Genet. 2021 Jan 7;11:595757. doi: 10.3389/fgene.2020.595757 (PMC7817997; doi:10.3389/fgene.2020.595757)
Supplement: Supplementary Table 1 — Clinical and demographic information of patients with IgAN in the GSE93798 dataset. [file Table_1.DOCX]

**Table S1** Clinical and demographic information of patients with IgAN in the GSE93798 dataset

| **Sample** | **Sex** | **Age,yr** | **Urine Albumin-to-Creatinine Ratio** | **Creatinine,μmol/L** | **eGFR** | **CKD Stage** | **BP** | **BP Medication** | **Steroids** | **Immuno suppressive Therapy** | **Oxford Classification** | | | | **Progression** |
| --- | --- | --- | --- | --- | --- | --- | --- | --- | --- | --- | --- | --- | --- | --- | --- |
|  |  |  |  |  |  |  |  |  |  |  | **M** | **E** | **S** | **T** |  |
| [**GSM2462561**](https://www.ncbi.nlm.nih.gov/geo/query/acc.cgi?acc=GSM2462561) | **Woman** | **54** | **229** | **132** | **39.4** | **3** | **130/80** | **Yes** | **No** | **No** | **0** | **0** | **1** | **1** | **-4.55** |
| [**GSM2462562**](https://www.ncbi.nlm.nih.gov/geo/query/acc.cgi?acc=GSM2462562) | **Man** | **46** | **323** | **155** | **45.6** | **3** | **150/90** | **Yes** | **No** | **No** | **1** | **1** | **1** | **1** | **0.6** |
| [**GSM2462564**](https://www.ncbi.nlm.nih.gov/geo/query/acc.cgi?acc=GSM2462564) | **Woman** | **45** | **155** | **132** | **40.5** | **3** | **140/80** | **Yes** | **No** | **No** | **1** | **0** | **1** | **1** | **-3.05** |
| [**GSM2462567**](https://www.ncbi.nlm.nih.gov/geo/query/acc.cgi?acc=GSM2462567) | **Woman** | **41** | **189** | **165** | **33** | **3** | **130/80** | **Yes** | **No** | **No** | **0** | **1** | **1** | **1** | **-5.37** |
| [**GSM2462556**](https://www.ncbi.nlm.nih.gov/geo/query/acc.cgi?acc=GSM2462556) | **Man** | **49** | **37** | **124** | **58.4** | **3** | **125/80** | **No** | **No** | **No** | **0** | **1** | **0** | **1** | **-3.86** |
| [**GSM2462573**](https://www.ncbi.nlm.nih.gov/geo/query/acc.cgi?acc=GSM2462573) | **Man** | **48** | **65** | **139** | **51.3** | **3** | **125/85** | **No** | **No** | **No** | **0** | **0** | **1** | **1** | **-18.99** |
| [**GSM2462560**](https://www.ncbi.nlm.nih.gov/geo/query/acc.cgi?acc=GSM2462560) | **Man** | **58** | **70** | **107** | **65.6** | **2** | **140/80** | **Yes** | **No** | **No** | **0** | **0** | **1** | **0** | **-8.49** |
| [**GSM2462565**](https://www.ncbi.nlm.nih.gov/geo/query/acc.cgi?acc=GSM2462565) | **Man** | **35** | **14** | **103** | **80.7** | **2** | **120/80** | **Yes** | **No** | **No** | **0** | **0** | **1** | **0** | **2.40** |
| [**GSM2462563**](https://www.ncbi.nlm.nih.gov/geo/query/acc.cgi?acc=GSM2462563) | **Man** | **58** | **61** | **117** | **67.9** | **2** | **130/90** | **Yes** | **No** | **No** | **0** | **0** | **1** | **2** | **-0.84** |
| [**GSM2462555**](https://www.ncbi.nlm.nih.gov/geo/query/acc.cgi?acc=GSM2462555) | **Man** | **34** | **5** | **124** | **64.9** | **2** | **126/85** | **Yes** | **No** | **No** | **0** | **0** | **1** | **1** | **1.18** |
| [**GSM2462566**](https://www.ncbi.nlm.nih.gov/geo/query/acc.cgi?acc=GSM2462566) | **Man** | **53** | **74** | **96** | **77.4** | **2** | **110/70** | **No** | **Yes** | **No** | **0** | **1** | **0** | **0** | **-27.74** |
| [**GSM2462571**](https://www.ncbi.nlm.nih.gov/geo/query/acc.cgi?acc=GSM2462571) | **Man** | **61** | **560** | **84** | **86** | **2** | **150/95** | **Yes** | **No** | **No** | **0** | **0** | **1** | **0** | **-3.94** |
| [**GSM2462572**](https://www.ncbi.nlm.nih.gov/geo/query/acc.cgi?acc=GSM2462572) | **Man** | **65** | **490** | **93** | **74** | **2** | **155/95** | **Yes** | **No** | **No** | **0** | **0** | **1** | **1** | **-1.88** |
| [**GSM2462557**](https://www.ncbi.nlm.nih.gov/geo/query/acc.cgi?acc=GSM2462557) | **Woman** | **17** | **15** | **71** | **108.2** | **1** | **130/80** | **No** | **No** | **No** | **1** | **1** | **1** | **0** | **-2.24** |
| [**GSM2462558**](https://www.ncbi.nlm.nih.gov/geo/query/acc.cgi?acc=GSM2462558) | **Man** | **30** | **75** | **91** | **97.1** | **1** | **130/90** | **Yes** | **No** | **No** | **0** | **0** | **1** | **0** | **4.49** |
| [**GSM2462559**](https://www.ncbi.nlm.nih.gov/geo/query/acc.cgi?acc=GSM2462559) | **Man** | **20** | **1.2** | **76** | **124.8** | **1** | **100/60** | **No** | **No** | **No** | **0** | **0** | **0** | **0** | **-6.94** |
| [**GSM2462568**](https://www.ncbi.nlm.nih.gov/geo/query/acc.cgi?acc=GSM2462568) | **Woman** | **23** | **0.6** | **68** | **109.3** | **1** | **110/65** | **No** | **No** | **No** | **0** | **0** | **0** | **0** | **3.92** |
| [**GSM2462570**](https://www.ncbi.nlm.nih.gov/geo/query/acc.cgi?acc=GSM2462570) | **Man** | **24** | **12** | **77** | **125.6** | **1** | **120/70** | **No** | **No** | **No** | **0** | **0** | **0** | **0** | **-17.45** |
| [**GSM2462574**](https://www.ncbi.nlm.nih.gov/geo/query/acc.cgi?acc=GSM2462574) | **Woman** | **23** | **13** | **66** | **113.3** | **1** | **120/70** | **No** | **No** | **No** | **0** | **1** | **1** | **0** | **3.77** |
