# Supplementary material for: Periostin Contributes to Immunoglobulin a Nephropathy by Promoting the Proliferation of Mesangial Cells: A Weighted Gene Correlation Network Analysis
Source: Front Genet. 2021 Jan 7;11:595757. doi: 10.3389/fgene.2020.595757 (PMC7817997; doi:10.3389/fgene.2020.595757)
Supplement: Supplementary Table 2 — Clinical and demographic information of the 35 IgAN patients in this study. [file Table_2.DOCX]

**Table S2** Clinical and demographic information of the 35 IgAN patients in this study

| **IgAN**  **sample** | **Sex** | **Age, yr** | **Creatinine**  **,μmol/L** | **eGFR** | **BUN,mmol/L** | **Cysc**  **,mg/L** | **Uric acid ,μmol/L** | **24U-pro**  **,mg/24h** | **Urine Albumin-to-Creatinine Ratio,mg/g** | **Oxford Classification** | | | | | **Lee's classification** |
| --- | --- | --- | --- | --- | --- | --- | --- | --- | --- | --- | --- | --- | --- | --- | --- |
|  |  |  |  |  |  |  |  |  |  | M | E | S | T | C |  |
| **IgAN 1** | **Man** | **20** | **445** | **15** | **21.4** | **4.57** | **307** | **3268.4** | **2854.7** | **1** | **1** | **1** | **2** | **1** | **Ⅴ** |
| **IgAN 2** | **Man** | **38** | **382** | **16** | **13.8** | **3.16** | **517** | **3914** | **1536.9** | **1** | **0** | **1** | **2** | **0** | **Ⅴ** |
| **IgAN 3** | **Man** | **52** | **294** | **20** | **17.5** | **2.84** | **539** | **5022** | **1584.1** | **1** | **0** | **1** | **2** | **1** | **Ⅴ** |
| **IgAN 4** | **Woman** | **52** | **158.5** | **32** | **6.1** | **2.05** | **338** | **816** | **892.1** | **1** | **0** | **1** | **1** | **1** | **Ⅳ** |
| **IgAN 5** | **Woman** | **33** | **160** | **36** | **6.6** | **1.96** | **423** | **4613.8** | **2402** | **1** | **0** | **1** | **1** | **1** | **Ⅳ** |
| **IgAN 6** | **Woman** | **48** | **137** | **39** | **9.6** | **1.65** | **445** | **1791.3** | **837** | **1** | **1** | **1** | **1** | **0** | **Ⅳ** |
| **IgAN 7** | **Man** | **45** | **172** | **40** | **5.8** | **1.74** | **360** | **1773.2** | **500.2** | **1** | **0** | **1** | **1** | **0** | **Ⅳ** |
| **IgAN 8** | **Man** | **28** | **154** | **52** | **7.3** | **1.46** | **472** | **3931.4** | **662.3** | **1** | **0** | **1** | **1** | **1** | **Ⅳ** |
| **IgAN 9** | **Woman** | **59** | **102** | **52** | **6.7** | **1.37** | **375** | **3909.4** | **2098.8** | **1** | **0** | **1** | **1** | **0** | **Ⅳ** |
| **IgAN 10** | **Man** | **24** | **151** | **55** | **5.2** | **1.17** | **410** | **2513** | **970.4** | **1** | **0** | **1** | **0** | **1** | **Ⅳ** |
| **IgAN 11** | **Man** | **43** | **136** | **55** | **7.05** | **1.41** | **456** | **2765.3** | **1453.6** | **1** | **0** | **1** | **1** | **1** | **Ⅳ** |
| **IgAN 12** | **Man** | **35** | **140** | **56** | **6.5** | **1.43** | **313** | **1304.8** | **946.1** | **1** | **0** | **1** | **1** | **0** | **Ⅳ** |
| **IgAN 13** | **Woman** | **49** | **100** | **57** | **5.8** | **0.93** | **209** | **681.6** | **303.2** | **1** | **0** | **1** | **1** | **0** | **Ⅳ** |
| **IgAN 14** | **Man** | **41** | **131** | **58** | **5.8** | **1.39** | **527** | **7657.6** | **1535.8** | **1** | **0** | **1** | **1** | **1** | **Ⅳ** |
| **IgAN 15** | **Woman** | **49** | **99** | **58** | **5.8** | **1.19** | **274** | **716** | **1021.7** | **1** | **0** | **1** | **1** | **1** | **Ⅳ** |
| **IgAN 16** | **Man** | **45** | **120.7** | **62** | **5.8** | **1.5** | **256** | **1042.8** | **540.9** | **1** | **0** | **1** | **1** | **1** | **Ⅳ** |
| **IgAN 17** | **Woman** | **42** | **97** | **62** | **3.1** | **1.14** | **342** | **3632** | **1920.9** | **1** | **0** | **1** | **1** | **1** | **Ⅳ** |
| **IgAN 18** | **Man** | **40** | **122** | **64** | **6** | **1.3** | **454** | **1911.6** | **282.9** | **1** | **1** | **1** | **0** | **0** | **Ⅲ-Ⅳ** |
| **IgAN 19** | **Man** | **27** | **127** | **66** | **5.5** | **1.16** | **489** | **3146.4** | **881.4** | **1** | **1** | **1** | **0** | **1** | **Ⅲ-Ⅳ** |
| **IgAN 20** | **Woman** | **36** | **96** | **66** | **4.8** | **1.07** | **318** | **538.5** | **270** | **1** | **0** | **1** | **0** | **0** | **Ⅳ** |
| **IgAN 21** | **Woman** | **43** | **90** | **67** | **3.9** | **1.14** | **361** | **2406.6** | **963.4** | **0** | **0** | **1** | **0** | **0** | **Ⅲ-Ⅳ** |
| **IgAN 22** | **Woman** | **64** | **79** | **69** | **4.9** | **1.04** | **320** | **1890** | **1015.7** | **0** | **0** | **0** | **0** | **0** | **Ⅲ-Ⅳ** |
| **IgAN 23** | **Man** | **28** | **121** | **70** | **4.7** | **1.16** | **413** | **233.1** | **95.1** | **1** | **0** | **1** | **1** | **0** | **Ⅳ** |
| **IgAN 24** | **Man** | **42** | **110.2** | **71** | **6** | **1.03** | **458** | **555** | **122.7** | **1** | **0** | **1** | **0** | **0** | **Ⅲ** |
| **IgAN 25** | **Woman** | **50** | **83** | **71** | **5.3** | **0.98** | **347** | **1453.5** | **578.7** | **1** | **0** | **1** | **1** | **1** | **Ⅳ** |
| **IgAN 26** | **Man** | **50** | **104** | **72** | **3.7** | **1.08** | **144** | **137.6** | **8.5** | **0** | **0** | **0** | **0** | **1** | **Ⅲ-Ⅳ** |
| **IgAN 27** | **Man** | **44** | **104** | **75** | **4.8** | **1.58** | **372** | **3741.4** | **525.6** | **1** | **1** | **1** | **0** | **0** | **Ⅲ-Ⅳ** |
| **IgAN 28** | **Man** | **55** | **98** | **75** | **5.1** | **1.07** | **294** | **697.4** | **279.5** | **1** | **0** | **0** | **0** | **0** | **Ⅲ** |
| **IgAN 29** | **Man** | **55** | **96.3** | **76** | **7.2** | **1.23** | **493** | **944** | **430.3** | **1** | **1** | **0** | **1** | **0** | **Ⅳ** |
| **IgAN 30** | **Woman** | **38** | **84** | **76** | **4.8** | **1.01** | **326** | **402.6** | **819.9** | **1** | **0** | **1** | **0** | **1** | **Ⅳ** |
| **IgAN 31** | **Woman** | **49** | **77.9** | **77** | **4.9** | **1.17** | **349** | **701** | **392** | **1** | **0** | **1** | **0** | **0** | **Ⅳ** |
| **IgAN 32** | **Man** | **28** | **108.4** | **80** | **4.5** | **1.25** | **435** | **3670.5** | **886.6** | **1** | **0** | **1** | **1** | **1** | **Ⅳ** |
| **IgAN 33** | **Woman** | **30** | **83** | **82** | **5.4** | **1.04** | **383** | **1282.6** | **356.2** | **1** | **0** | **1** | **1** | **1** | **Ⅳ** |
| **IgAN 34** | **Woman** | **47** | **75** | **82** | **6.5** | **1.04** | **369** | **1520.2** | **529** | **0** | **1** | **1** | **1** | **1** | **Ⅳ** |
| **IgAN 35** | **Man** | **31** | **103** | **83** | **4.9** | **1.11** | **312** | **1251** | **575.6** | **1** | **0** | **1** | **1** | **1** | **Ⅳ** |
