# Supplementary material for: Periostin Contributes to Immunoglobulin a Nephropathy by Promoting the Proliferation of Mesangial Cells: A Weighted Gene Correlation Network Analysis
Source: Front Genet. 2021 Jan 7;11:595757. doi: 10.3389/fgene.2020.595757 (PMC7817997; doi:10.3389/fgene.2020.595757)
Supplement: Supplementary Table 3 — Clinical and demographic information of the 15 healthy control subjects in this study. [file Table_3.DOCX]

| **Healthy control sample** | **sex** | **Age,yr** | **Creatinine**  **,μmol/L** | **eGFR** | **BUN,mmol/L** | **Cysc,mg/L** | **Uric acid**  **,μmol/L** | **24U-pro**  **,mg/24h** | **Urine Albumin-to-Creatinine Ratio,mg/g** |
| --- | --- | --- | --- | --- | --- | --- | --- | --- | --- |
| **HC 1** | **Man** | **45** | **45** | **130** | **3.4** | **0.56** | **245** | **145** | **25.4** |
| **HC 2** | **Man** | **56** | **45** | **120** | **4.3** | **0.45** | **324** | **123** | **17.8** |
| **HC 3** | **Woman** | **67** | **43** | **101** | **5.1** | **0.54** | **325** | **143** | **23.5** |
| **HC 4** | **Man** | **65** | **41** | **117** | **3.5** | **0.43** | **245** | **124** | **24.6** |
| **HC 5** | **Woman** | **63** | **56** | **95** | **4.2** | **0.82** | **267** | **145** | **18.6** |
| **HC 6** | **Man** | **57** | **56** | **107** | **3.6** | **0.46** | **278** | **146** | **18.9** |
| **HC 7** | **Man** | **58** | **36** | **130** | **4.6** | **0.78** | **327** | **150** | **15.6** |
| **HC 8** | **Woman** | **52** | **37** | **118** | **4.3** | **0.58** | **347** | **132** | **20.4** |
| **HC 9** | **Woman** | **61** | **46** | **103** | **4.1** | **0.67** | **452** | **143** | **26.3** |
| **HC 10** | **Man** | **53** | **65** | **106** | **5.1** | **0.76** | **367** | **138** | **24.6** |
| **HC 11** | **Man** | **49** | **54** | **117** | **4.8** | **0.61** | **387** | **149** | **21.4** |
| **HC 12** | **Woman** | **70** | **56** | **91** | **3.9** | **1.01** | **365** | **138** | **23.6** |
| **HC 13** | **Man** | **67** | **75** | **90** | **3.8** | **0.69** | **289** | **125** | **22.5** |
| **HC 14** | **Man** | **59** | **47** | **116** | **3.6** | **0.68** | **295** | **120** | **27.4** |
| **HC 15** | **Woman** | **60** | **61** | **95** | **3.7** | **1.02** | **361** | **132** | **25.6** |

**Table S3** Clinical and demographic information of the 15 healthy control subjects in this study
