# Supplementary material for: Periostin Contributes to Immunoglobulin a Nephropathy by Promoting the Proliferation of Mesangial Cells: A Weighted Gene Correlation Network Analysis
Source: Front Genet. 2021 Jan 7;11:595757. doi: 10.3389/fgene.2020.595757 (PMC7817997; doi:10.3389/fgene.2020.595757)
Supplement: Supplementary Table 4 — DEGs in dataset GSE37460. [file Table_4.DOCX]

**Table S4** DEGs in dataset GSE37460

| **Gene symbol** | **logFC** | **AveExpr** | **t** | **P.Value** | **adj.P.Val** | **B** |
| --- | --- | --- | --- | --- | --- | --- |
| **CDH5** | 1.552352723 | 9.059642029 | 8.810023997 | 1.28E-10 | 2.56E-07 | 14.08536641 |
| **COL4A3BP** | 1.020623238 | 6.733354508 | 4.415497914 | 8.43E-05 | 0.000905193 | 1.348334484 |
| **TSPAN2** | 1.062650568 | 7.912157564 | 3.53015035 | 0.001130546 | 0.005674113 | -1.09118365 |
| **CDH13** | 1.061065704 | 8.888634233 | 3.555389011 | 0.001052768 | 0.005399725 | -1.024831614 |
| **LPAR6** | 1.08200333 | 10.32449637 | 7.456817564 | 7.02E-09 | 4.87E-06 | 10.29696148 |
| **RCAN2** | 1.17225988 | 10.34341681 | 6.225380591 | 3.11E-07 | 4.12E-05 | 6.685454266 |
| **KLF2** | 1.121279954 | 10.42502054 | 4.294100407 | 0.000121529 | 0.001157328 | 1.002437591 |
| **CD52** | 1.071371199 | 7.403202471 | 2.836466299 | 0.00735492 | 0.022814367 | -2.813361904 |
| **POSTN** | 1.432416223 | 10.93084866 | 4.589268447 | 4.98E-05 | 0.000658987 | 1.848113092 |
| **PLK2** | 1.023211197 | 8.194383171 | 4.25826379 | 0.000135317 | 0.001240184 | 0.90089336 |
| **FGL2** | 1.49291306 | 10.10141766 | 6.525146346 | 1.22E-07 | 2.66E-05 | 7.574177557 |
| **IFI44L** | 1.185281233 | 8.200998172 | 2.478913071 | 0.017859468 | 0.045498183 | -3.608715486 |
| **TMSB15A** | 1.037597981 | 9.553104865 | 2.969439913 | 0.00521063 | 0.017645625 | -2.50009108 |
| **RAPGEF4** | 1.544759441 | 7.34864945 | 7.844863322 | 2.18E-09 | 1.74E-06 | 11.40633857 |
| **VSIG4** | 1.034838309 | 7.89182041 | 2.301011312 | 0.02712305 | 0.06364897 | -3.976321441 |
| **COL1A2** | 1.199459246 | 8.746935938 | 2.64393376 | 0.01194836 | 0.033259097 | -3.250572794 |
| **COL4A1** | 1.289359053 | 10.92710325 | 7.376882224 | 8.95E-09 | 5.10E-06 | 10.06640428 |
| **COL6A3** | 1.363632558 | 7.669465257 | 2.656095181 | 0.01159357 | 0.03249079 | -3.223557143 |
| **COL15A1** | 1.037987751 | 7.07553577 | 2.710580614 | 0.010120368 | 0.029239063 | -3.101508441 |
| **CFD** | 1.007326037 | 7.353987358 | 3.312214849 | 0.002074655 | 0.008787165 | -1.654302241 |
| **AGTR1** | 1.215635085 | 9.222869303 | 4.969823501 | 1.55E-05 | 0.000321255 | 2.957940947 |
| **ECM1** | 1.336145285 | 10.16254169 | 3.228222656 | 0.002610386 | 0.01044591 | -1.866314435 |
| **S1PR1** | 1.104918864 | 9.15197811 | 5.090173505 | 1.07E-05 | 0.000263868 | 3.31228503 |
| **EDNRB** | 1.376504005 | 7.274342848 | 3.652980142 | 0.000797751 | 0.004444294 | -0.766191609 |
| **MECOM** | 1.759858032 | 7.114919723 | 5.429020419 | 3.72E-06 | 0.000153085 | 4.315669392 |
| **F8** | 1.144741315 | 10.05976967 | 6.089736842 | 4.74E-07 | 5.25E-05 | 6.282161391 |
| **FCN1** | 1.104586425 | 8.453004814 | 2.460404437 | 0.01866726 | 0.047035831 | -3.647877072 |
| **CD93** | 1.122248645 | 9.093177333 | 6.268497288 | 2.72E-07 | 3.90E-05 | 6.813523319 |
| **FOXO3** | 1.326214884 | 8.409683091 | 4.158381804 | 0.000182347 | 0.001524147 | 0.619336265 |
| **FN1** | 1.033271616 | 8.3177039 | 3.03002465 | 0.004442537 | 0.015596473 | -2.35447881 |
| **HMHA1** | 1.066278985 | 8.666052782 | 6.205326219 | 3.31E-07 | 4.21E-05 | 6.625864584 |
| **LY96** | 1.410177724 | 7.30839176 | 5.354959415 | 4.69E-06 | 0.000164776 | 4.095798269 |
| **SOSTDC1** | 1.24285443 | 8.106526654 | 3.885228906 | 0.000407962 | 0.00271883 | -0.138380005 |
| **RWDD3** | 1.073472291 | 6.537609738 | 5.855180533 | 9.85E-07 | 8.09E-05 | 5.583876506 |
| **GATA3** | 1.183854271 | 7.985318754 | 6.339586904 | 2.18E-07 | 3.43E-05 | 7.024522533 |
| **GBP2** | 1.076319588 | 8.946956518 | 6.0285292 | 5.74E-07 | 5.92E-05 | 6.100024625 |
| **TSPAN13** | 1.020814865 | 9.578781184 | 4.776042272 | 2.81E-05 | 0.000462273 | 2.390516051 |
| **SLCO3A1** | 1.21246117 | 7.662693203 | 6.352062536 | 2.09E-07 | 3.43E-05 | 7.061529247 |
| **CNIH4** | 1.209720009 | 8.572925424 | 6.259757829 | 2.79E-07 | 3.90E-05 | 6.787570123 |
| **PYCARD** | 1.131257831 | 7.742670582 | 4.089804355 | 0.00022354 | 0.001766039 | 0.427340924 |
| **HBA2** | 3.553117694 | 12.06980329 | 7.26119091 | 1.27E-08 | 6.86E-06 | 9.731571437 |
| **HBB** | 3.17214727 | 11.19713547 | 6.302372027 | 2.44E-07 | 3.66E-05 | 6.91409172 |
| **HCLS1** | 1.10492492 | 9.593180539 | 5.01558973 | 1.34E-05 | 0.000295838 | 3.092533412 |
| **HLA-DQB1** | 1.044983721 | 9.841434014 | 3.697697434 | 0.000701899 | 0.004059676 | -0.646616806 |
| **HLX** | 1.216152414 | 8.487456138 | 6.644162486 | 8.47E-08 | 2.26E-05 | 7.925726458 |
| **HOXA7** | 1.011337841 | 6.793314745 | 4.15539635 | 0.000183975 | 0.001532398 | 0.610954982 |
| **HTR2B** | 1.413193686 | 6.786578583 | 3.514053201 | 0.001183007 | 0.005864216 | -1.133384312 |
| **ID1** | 1.178851585 | 10.65116876 | 5.773819396 | 1.27E-06 | 9.16E-05 | 5.341562359 |
| **IL10RA** | 1.029289426 | 7.756381653 | 3.117645803 | 0.00351838 | 0.013080176 | -2.140840391 |
| **IL13RA2** | 1.022863349 | 11.34125394 | 3.009513387 | 0.004689768 | 0.016292277 | -2.403972856 |
| **ITGB2** | 1.156373782 | 7.870179471 | 3.369502851 | 0.00177131 | 0.007864378 | -1.508047531 |
| **KDR** | 1.208370562 | 10.53174522 | 4.746482164 | 3.08E-05 | 0.000489332 | 2.304351349 |
| **TNPO1** | 1.147787312 | 9.428765149 | 4.2679133 | 0.00013146 | 0.00121797 | 0.928209142 |
| **LTF** | 1.217728327 | 8.199522725 | 2.717111202 | 0.009955931 | 0.02887558 | -3.086769736 |
| **MATN2** | 1.14133209 | 9.449793107 | 6.536962036 | 1.18E-07 | 2.66E-05 | 7.609116209 |
| **NCF2** | 1.012119366 | 8.040017315 | 2.82995361 | 0.007478675 | 0.023101986 | -2.828474553 |
| **GIMAP6** | 1.103406278 | 10.25465408 | 4.863461389 | 2.15E-05 | 0.000398247 | 2.645968076 |
| **NNMT** | 1.185642328 | 7.51666121 | 2.365264397 | 0.023368363 | 0.056216611 | -3.84584928 |
| **NPY1R** | 1.062283698 | 9.917020684 | 5.325609771 | 5.13E-06 | 0.000173942 | 4.008738385 |
| **GOLT1B** | 1.026835172 | 7.358042296 | 5.747112143 | 1.38E-06 | 9.67E-05 | 5.262025704 |
| **GLTP** | 1.193240016 | 6.672622226 | 5.82730132 | 1.07E-06 | 8.63E-05 | 5.500844803 |
| **PCDH12** | 1.10757492 | 9.061615107 | 5.899383597 | 8.58E-07 | 7.57E-05 | 5.715519361 |
| **MS4A4A** | 1.010793419 | 5.924519999 | 2.048418337 | 0.047660624 | 0.098287711 | -4.462664045 |
| **PCYOX1** | 1.007966289 | 7.435814943 | 3.188404015 | 0.002908132 | 0.01134513 | -1.965785704 |
| **PDGFRA** | 1.336710925 | 9.008859602 | 5.577109591 | 2.35E-06 | 0.000124753 | 4.755946219 |
| **KLF13** | 1.082898283 | 6.554022677 | 5.244922536 | 6.60E-06 | 0.00020169 | 3.769641232 |
| **PECAM1** | 1.305287209 | 9.909293336 | 6.386124359 | 1.88E-07 | 3.27E-05 | 7.16253194 |
| **FXYD6** | 1.102153139 | 10.3111038 | 5.988024304 | 6.51E-07 | 6.54E-05 | 5.979453884 |
| **EXOSC10** | 1.129299347 | 7.652999597 | 4.97752067 | 1.51E-05 | 0.000316455 | 2.980563158 |
| **SOX18** | 1.161483643 | 6.138122087 | 5.603361664 | 2.16E-06 | 0.000120775 | 4.834061912 |
| **MPHOSPH8** | 1.067566128 | 8.165042264 | 4.968947125 | 1.55E-05 | 0.000321497 | 2.955365622 |
| **XAF1** | 1.071221999 | 7.523052639 | 3.281204422 | 0.002258933 | 0.009362125 | -1.73291868 |
| **DPP8** | 1.02366165 | 10.10240276 | 4.219461066 | 0.000151977 | 0.001346513 | 0.79125156 |
| **RCBTB1** | 1.042635781 | 8.41338151 | 6.4271105 | 1.66E-07 | 3.10E-05 | 7.283994902 |
| **GIMAP4** | 1.177784109 | 10.55796008 | 5.859240054 | 9.73E-07 | 8.09E-05 | 5.59596669 |
| **C8orf4** | 1.834724813 | 9.574594235 | 11.4143524 | 1.10E-13 | 1.32E-09 | 20.65337353 |
| **KIAA1462** | 1.178024458 | 7.73631875 | 4.826093168 | 2.41E-05 | 0.000421629 | 2.536660093 |
| **PTPRB** | 1.046827211 | 8.805374698 | 3.739549845 | 0.000622339 | 0.00371806 | -0.534120946 |
| **RALA** | 1.389771048 | 6.887841259 | 5.416756272 | 3.87E-06 | 0.000153085 | 4.279242934 |
| **ACTA2** | 1.355590822 | 10.88535578 | 5.575530423 | 2.36E-06 | 0.000124753 | 4.751247774 |
| **SOX17** | 1.398297591 | 6.712229846 | 7.914777671 | 1.77E-09 | 1.60E-06 | 11.60437779 |
| **ST3GAL1** | 1.091891502 | 7.757937959 | 3.547841563 | 0.00107547 | 0.005493429 | -1.044697348 |
| **SLC14A1** | 1.359511839 | 8.444283768 | 4.51287923 | 6.28E-05 | 0.0007568 | 1.627781573 |
| **SLCO2A1** | 1.159555924 | 10.78506775 | 4.356017452 | 0.000100881 | 0.001025177 | 1.178498679 |
| **SNAI2** | 1.274023789 | 9.589178625 | 6.341562816 | 2.16E-07 | 3.43E-05 | 7.030384173 |
| **TCF4** | 1.188092524 | 6.814730015 | 3.983309117 | 0.000306105 | 0.002221881 | 0.131473426 |
| **TGFBI** | 1.033497645 | 9.09778333 | 2.737175664 | 0.009466085 | 0.027839646 | -3.041341213 |
| **C1QA** | 1.647305039 | 7.662639505 | 3.539302117 | 0.001101725 | 0.005581418 | -1.067149883 |
| **C1QB** | 1.596988869 | 7.381863251 | 3.038352017 | 0.004345714 | 0.015324023 | -2.334328033 |
| **PHLDA2** | 1.228365682 | 6.270189758 | 3.574609348 | 0.000997018 | 0.005178336 | -0.974151363 |
| **TYROBP** | 1.433864429 | 8.618173788 | 3.685843875 | 0.000726163 | 0.004151836 | -0.678376712 |
| **ZNF148** | 1.070636681 | 7.108647884 | 4.424849358 | 8.20E-05 | 0.000893598 | 1.375096117 |
| **NETO2** | 1.498491956 | 7.860988326 | 5.710944636 | 1.54E-06 | 0.000100784 | 5.154324284 |
| **DYSF** | 1.113336759 | 10.56016054 | 5.64792564 | 1.88E-06 | 0.000110219 | 4.966700294 |
| **SRPX** | 1.042160747 | 7.127189499 | 4.254518478 | 0.000136844 | 0.00124575 | 0.890296453 |
| **MAGT1** | 1.496877812 | 8.069826051 | 5.174608604 | 8.21E-06 | 0.000230152 | 3.561615344 |
| **FCN3** | 1.074019698 | 11.58531639 | 3.413354274 | 0.001568274 | 0.007172647 | -1.395223522 |
| **PPAP2B** | 1.214924222 | 11.12246007 | 4.384658438 | 9.25E-05 | 0.000959938 | 1.260194177 |
| **HYAL2** | 1.056538791 | 9.910179762 | 6.769473645 | 5.75E-08 | 1.86E-05 | 8.294886184 |
| **ARHGEF6** | 1.035591141 | 9.036024604 | 7.249321426 | 1.32E-08 | 6.86E-06 | 9.697144485 |
| **GMFG** | 1.331397679 | 8.959294476 | 6.747181547 | 6.16E-08 | 1.92E-05 | 8.229293901 |
| **CD53** | 1.04995358 | 9.722248685 | 3.110757108 | 0.003583875 | 0.013242734 | -2.157763775 |
| **ISG15** | 1.049645795 | 9.546434613 | 2.845554053 | 0.007185426 | 0.022387311 | -2.792237151 |
| **CD97** | 1.096187154 | 8.315328995 | 6.030260957 | 5.71E-07 | 5.92E-05 | 6.105178898 |
| **TRIB1** | -1.491711965 | 8.255223581 | -5.384652304 | 4.27E-06 | 0.000158683 | 4.183919928 |
| **HRSP12** | -1.104862449 | 9.215028863 | -3.388911412 | 0.001678523 | 0.007556195 | -1.458203789 |
| **GLYAT** | -1.372095951 | 9.168020276 | -3.422277717 | 0.001529781 | 0.007059301 | -1.37217396 |
| **SLC17A3** | -1.064539879 | 9.536682249 | -2.328758855 | 0.02543964 | 0.060400027 | -3.920302144 |
| **ALDH1L1** | -1.031375018 | 8.717257269 | -2.608513058 | 0.013039548 | 0.035584311 | -3.328779683 |
| **FTCD** | -1.351917778 | 8.288068539 | -3.969987465 | 0.000318325 | 0.002287015 | 0.094668156 |
| **SLC27A2** | -1.355433435 | 9.30019277 | -2.975554575 | 0.005127782 | 0.017454012 | -2.485474796 |
| **SLC7A9** | -1.37404893 | 9.919865547 | -2.860114523 | 0.006921449 | 0.021797643 | -2.758302238 |
| **SUPT16H** | -1.12233501 | 9.999968259 | -7.778170966 | 2.67E-09 | 1.99E-06 | 11.21688733 |
| **CISH** | -1.016934871 | 8.5939087 | -5.513064172 | 2.86E-06 | 0.000139459 | 4.565448333 |
| **SIK1** | -2.346134951 | 7.561520538 | -8.943928498 | 8.74E-11 | 2.48E-07 | 14.44718161 |
| **CYP27B1** | -2.309864818 | 8.056781774 | -8.032017259 | 1.25E-09 | 1.25E-06 | 11.93514606 |
| **AFM** | -1.105737784 | 6.898236458 | -3.270612309 | 0.002325376 | 0.009561299 | -1.759680854 |
| **DPYS** | -1.192578066 | 8.82375325 | -2.554046543 | 0.014897443 | 0.039465851 | -3.447631286 |
| **AGT** | -1.097415972 | 7.843369716 | -2.8979699 | 0.006276954 | 0.020258741 | -2.669569711 |
| **EGR1** | -1.465114905 | 8.705837432 | -5.150753197 | 8.84E-06 | 0.000239678 | 3.491116908 |
| **EGR2** | -1.245315226 | 6.700681561 | -2.589464361 | 0.013663611 | 0.036916751 | -3.370541364 |
| **EGR3** | -1.402932959 | 6.53626771 | -3.354874043 | 0.001844467 | 0.008090089 | -1.545518622 |
| **ALB** | -2.551299538 | 7.276346735 | -4.645893499 | 4.19E-05 | 0.0005953 | 2.012028858 |
| **FABP1** | -1.0984944 | 7.324101442 | -2.513326168 | 0.01644174 | 0.042557813 | -3.535352454 |
| **FBP1** | -1.211960032 | 10.24513365 | -3.339755759 | 0.001923103 | 0.00834624 | -1.584154489 |
| **SYNE2** | -1.081983129 | 8.833932077 | -5.488278323 | 3.09E-06 | 0.000142481 | 4.491758238 |
| **FOS** | -1.188835732 | 8.688825221 | -2.37558681 | 0.022810967 | 0.05505286 | -3.824643246 |
| **FOSB** | -3.341766962 | 7.494501371 | -9.816772788 | 7.55E-12 | 4.51E-08 | 16.74271588 |
| **G6PC** | -1.114683087 | 5.658903924 | -3.14175755 | 0.003297948 | 0.012475257 | -2.081437472 |
| **GHR** | -1.007894323 | 10.63338424 | -5.447315976 | 3.51E-06 | 0.000150626 | 4.37002154 |
| **GK** | -1.079402694 | 7.966286375 | -4.044691517 | 0.000255455 | 0.001954891 | 0.301657966 |
| **GSTA1** | -1.635867298 | 10.26326078 | -3.038754085 | 0.00434109 | 0.015312232 | -2.333354275 |
| **NR4A1** | -1.325952802 | 8.385455378 | -8.528508345 | 2.90E-10 | 4.16E-07 | 13.31661685 |
| **HPD** | -1.490040135 | 9.03146323 | -2.621076732 | 0.012642416 | 0.03476624 | -3.30112157 |
| **APOD** | -1.269297949 | 10.66012299 | -2.484919789 | 0.017604241 | 0.044963555 | -3.595961505 |
| **IGF1** | -1.089669577 | 8.160322507 | -3.281234488 | 0.002258747 | 0.009362125 | -1.732842649 |
| **IGFBP1** | -1.161332861 | 6.348828466 | -2.241788239 | 0.031054677 | 0.070657217 | -4.094209001 |
| **APOH** | -1.599830799 | 6.798838274 | -5.725415397 | 1.48E-06 | 0.000100533 | 5.197414583 |
| **KNG1** | -1.183726365 | 8.836744633 | -2.241938605 | 0.031044081 | 0.070653357 | -4.09391261 |
| **ALDH6A1** | -1.173824973 | 9.341034872 | -3.166797014 | 0.003082925 | 0.011879788 | -2.019475307 |
| **MT1G** | -1.186524426 | 12.08204496 | -3.756090344 | 0.000593366 | 0.003584469 | -0.489509814 |
| **ATF3** | -2.260742787 | 8.786199622 | -7.431210997 | 7.59E-09 | 4.87E-06 | 10.22317653 |
| **NFIL3** | -1.161818473 | 8.549887512 | -6.157176689 | 3.84E-07 | 4.64E-05 | 6.4827395 |
| **NR4A2** | -1.56229012 | 5.571102474 | -8.065203067 | 1.13E-09 | 1.23E-06 | 12.02846686 |
| **PAH** | -1.002858968 | 10.80372515 | -2.238721536 | 0.031271487 | 0.071082908 | -4.100250572 |
| **SERPINA5** | -1.071301782 | 8.944461082 | -2.140890698 | 0.038935496 | 0.083941506 | -4.289667908 |
| **PCK1** | -1.383859169 | 10.6604668 | -2.915822855 | 0.005992912 | 0.019579683 | -2.62747177 |
| **HAO2** | -1.443899382 | 9.109771323 | -2.950445715 | 0.005476082 | 0.01832664 | -2.545378125 |
| **UPB1** | -1.264628559 | 7.485189413 | -3.23385978 | 0.002570653 | 0.010322489 | -1.852177539 |
| **SERPINA1** | -1.291844107 | 9.832523765 | -3.846577693 | 0.00045657 | 0.002961515 | -0.243987047 |
| **PLG** | -1.168485396 | 9.02760318 | -2.064928683 | 0.045987352 | 0.095628428 | -4.432216319 |
| **RIPK4** | -1.155817957 | 7.921586565 | -5.04338837 | 1.23E-05 | 0.000282553 | 3.174381242 |
| **CYCS** | -1.036362474 | 10.1510195 | -4.740307728 | 3.14E-05 | 0.000495138 | 2.286367854 |
| **SLC22A11** | -1.034313121 | 7.958077693 | -3.05829424 | 0.004121875 | 0.014677506 | -2.285939327 |
| **APOM** | -1.250406681 | 9.114313449 | -2.971080856 | 0.005188274 | 0.017594825 | -2.496170404 |
| **AZGP1** | -1.169649123 | 7.373095082 | -2.243925824 | 0.030904349 | 0.070390013 | -4.089994152 |
| **DNAJC12** | -1.099802587 | 7.980267414 | -3.673739806 | 0.000751777 | 0.004264847 | -0.710760923 |
| **PSG5** | -1.191161147 | 7.066126584 | -6.052130577 | 5.33E-07 | 5.69E-05 | 6.17026503 |
| **GBA3** | -1.135654087 | 8.025412761 | -3.186751565 | 0.00292116 | 0.011377401 | -1.969898936 |
| **PRODH2** | -1.224670465 | 10.29153703 | -3.486902274 | 0.001276837 | 0.006215812 | -1.204351787 |
| **ACE2** | -1.073387102 | 7.413174936 | -2.54433142 | 0.015253307 | 0.04017723 | -3.468648913 |
| **RBP4** | -1.503149019 | 7.671206292 | -2.833610568 | 0.007408948 | 0.022934374 | -2.819991315 |
| **PBLD** | -1.178126374 | 9.421179714 | -3.625864702 | 0.000861903 | 0.004671092 | -0.83837821 |
| **SLC13A3** | -1.065365184 | 9.150678132 | -2.719560931 | 0.009894889 | 0.028754318 | -3.081234991 |
| **SLC2A2** | -1.449625298 | 6.560089784 | -4.299643963 | 0.000119523 | 0.001141861 | 1.018169209 |
| **SLC10A2** | -1.02141606 | 7.201454832 | -3.227197217 | 0.002617676 | 0.010470535 | -1.868884601 |
| **SLC17A1** | -1.057718477 | 7.597580598 | -2.686835298 | 0.010739701 | 0.030628891 | -3.154900987 |
| **TFRC** | -1.071044067 | 9.060853464 | -5.378738469 | 4.35E-06 | 0.000160139 | 4.166365676 |
| **KLF10** | -1.007394187 | 10.47383143 | -6.017555621 | 5.94E-07 | 6.02E-05 | 6.067362396 |
| **UMOD** | -1.932676382 | 10.79751351 | -2.54548426 | 0.015210675 | 0.04009932 | -3.466157769 |
| **XPNPEP2** | -1.264430507 | 7.821286788 | -2.81719434 | 0.007726778 | 0.023720616 | -2.858018445 |
| **CRISPLD2** | -1.203473314 | 8.255596506 | -3.686392519 | 0.000725023 | 0.004147296 | -0.676907701 |
| **ARID5B** | -1.728444452 | 9.754701516 | -6.948540478 | 3.31E-08 | 1.28E-05 | 8.820425695 |
| **CMAH** | -1.03573809 | 8.377270186 | -3.947854094 | 0.000339686 | 0.002388587 | 0.033622234 |
| **SERPINA6** | -1.228765466 | 7.312265038 | -3.454809072 | 0.001396974 | 0.006638543 | -1.287888885 |
| **ZNF160** | -1.02924176 | 5.915684518 | -4.245382896 | 0.000140639 | 0.001269563 | 0.864460954 |
| **SLC22A6** | -1.104164009 | 7.950999497 | -2.518905567 | 0.016221823 | 0.042189437 | -3.523391259 |
| **SLC22A8** | -1.770381656 | 8.008869277 | -4.319463222 | 0.000112612 | 0.00109547 | 1.074463881 |
| **ACY1** | -1.091683834 | 10.50124246 | -2.815506009 | 0.007760177 | 0.023793765 | -2.861921389 |
| **GDF15** | -1.406852915 | 7.053575515 | -3.987570505 | 0.000302293 | 0.002208751 | 0.143256738 |
| **CXCL14** | -1.134964481 | 10.5747387 | -2.439885267 | 0.019601572 | 0.048834493 | -3.691048451 |
