# Supplementary material for: Periostin Contributes to Immunoglobulin a Nephropathy by Promoting the Proliferation of Mesangial Cells: A Weighted Gene Correlation Network Analysis
Source: Front Genet. 2021 Jan 7;11:595757. doi: 10.3389/fgene.2020.595757 (PMC7817997; doi:10.3389/fgene.2020.595757)
Supplement: Supplementary Table 5 — DEGs in dataset GSE104948. [file Table_5.DOCX]

**Table S5** DEGs in dataset GSE104948

| **Gene symbol** | **logFC** | **AveExpr** | **t** | **P.Value** | **adj.P.Val** | **B** |
| --- | --- | --- | --- | --- | --- | --- |
| **ARPC1B** | 1.360885162 | 10.36448289 | 4.885782955 | 3.04E-05 | 1.18E-02 | 2.421870674 |
| **LPAR6** | 1.450233058 | 10.442624 | 6.371822266 | 4.46E-07 | 0.000672562 | 6.253895084 |
| **CD52** | 1.887398653 | 7.439724943 | 3.22686704 | 0.002970322 | 0.102761217 | -1.731900062 |
| **IFI30** | 2.149995268 | 11.2166439 | 4.82860117 | 3.58E-05 | 0.012010107 | 2.273374987 |
| **IFI44** | 1.472088714 | 9.380521976 | 3.372721136 | 2.03E-03 | 8.38E-02 | -1.389266322 |
| **POSTN** | 1.421473838 | 11.14037014 | 2.661508353 | 1.23E-02 | 2.04E-01 | -2.98632081 |
| **HCP5** | 1.131826817 | 9.19820908 | 3.666903433 | 0.000921908 | 0.058896643 | -0.679628048 |
| **FGL2** | 1.320446064 | 10.29808521 | 5.138447993 | 1.48E-05 | 0.006615243 | 3.078767183 |
| **IFI44L** | 1.540730494 | 8.328950322 | 2.077402699 | 4.62E-02 | 0.352280749 | -4.122030393 |
| **GLIPR1** | 1.176642928 | 7.234930791 | 2.851764664 | 0.007705281 | 0.165855275 | -2.578804045 |
| **TMSB15A** | 1.838050816 | 9.547429812 | 3.726683169 | 7.84E-04 | 5.38E-02 | -0.532782777 |
| **ADAMTS5** | 1.300642256 | 7.088110997 | 2.975984254 | 0.005648774 | 0.144775357 | -2.304312962 |
| **PRSS23** | 1.356152562 | 10.18305852 | 2.629818072 | 0.013226327 | 0.208820308 | -3.052577457 |
| **CORO1A** | 1.226272969 | 7.320316783 | 3.304563472 | 2.42E-03 | 9.15E-02 | -1.550211558 |
| **CD300A** | 1.184994049 | 7.0133043 | 2.950012942 | 0.006030321 | 0.148895893 | -2.362220859 |
| **COL1A2** | 2.256204517 | 8.841113599 | 2.929958974 | 0.006341462 | 0.153440504 | -2.406750458 |
| **COL3A1** | 1.834150781 | 10.32634563 | 4.334073686 | 1.45E-04 | 2.29E-02 | 0.996635101 |
| **COL4A2** | 1.002280572 | 9.97635417 | 3.227181588 | 0.002967893 | 0.102761217 | -1.731168473 |
| **COL6A3** | 2.050548378 | 7.810978951 | 2.304269178 | 0.028137242 | 0.288613415 | -3.703583093 |
| **CSF1R** | 1.396328336 | 8.417158236 | 3.10678447 | 0.004050392 | 0.121225366 | -2.008723046 |
| **CSF2RB** | 1.261373678 | 8.584319632 | 2.40914132 | 2.22E-02 | 0.261881445 | -3.500027357 |
| **CSTA** | 2.084641381 | 6.767904999 | 4.167987355 | 0.000231687 | 0.030406402 | 0.572822805 |
| **CTSS** | 1.707389595 | 7.655448702 | 3.598433664 | 1.11E-03 | 0.063586715 | -0.846794681 |
| **CX3CR1** | 3.132192485 | 8.709849726 | 6.596553462 | 2.38E-07 | 0.000603163 | 6.819161798 |
| **CYBB** | 1.532654741 | 6.933826678 | 2.868036802 | 7.40E-03 | 0.163651966 | -2.543210128 |
| **CFD** | 1.033154007 | 7.415769529 | 2.078486298 | 4.61E-02 | 3.52E-01 | -4.120106558 |
| **DOCK2** | 1.541909379 | 6.742175219 | 3.759388986 | 0.000716847 | 0.051807147 | -0.452106793 |
| **APLNR** | 1.595903984 | 10.66681863 | 4.536156125 | 8.22E-05 | 1.74E-02 | 1.516240308 |
| **APOBEC3A** | 1.399958156 | 6.904207151 | 2.3333476 | 0.026354596 | 0.280117022 | -3.647758053 |
| **EMP1** | 1.106515789 | 8.332418621 | 2.663674801 | 0.012195332 | 0.203903015 | -2.981773776 |
| **MECOM** | 1.201573004 | 7.769111426 | 3.091560116 | 4.21E-03 | 1.24E-01 | -2.043456197 |
| **EVI2A** | 1.441098425 | 7.156738716 | 2.332758247 | 2.64E-02 | 0.280117022 | -3.648894249 |
| **EVI2B** | 1.651512898 | 7.102283286 | 2.812789417 | 0.008483858 | 0.172946727 | -2.663599023 |
| **FCER1G** | 2.331024157 | 9.01540843 | 5.381054736 | 7.40E-06 | 4.24E-03 | 3.709362976 |
| **FCGR3B** | 1.850548765 | 8.746890056 | 2.975224984 | 5.66E-03 | 1.45E-01 | -2.306009685 |
| **FCN1** | 2.923510264 | 8.404253082 | 5.298565269 | 9.36E-06 | 4.52E-03 | 3.495069259 |
| **FGR** | 1.203818957 | 8.438312939 | 3.374520615 | 2.02E-03 | 0.083685347 | -1.384998028 |
| **FHL2** | 1.163647267 | 9.526287218 | 2.969098457 | 5.75E-03 | 1.46E-01 | -2.319692182 |
| **FN1** | 1.400642184 | 8.446943454 | 2.357312505 | 2.50E-02 | 2.75E-01 | -3.601391808 |
| **FPR1** | 1.017560275 | 7.386854892 | 2.193630163 | 0.035957976 | 0.320477043 | -3.911519267 |
| **LY96** | 1.650033919 | 7.572700938 | 4.389714539 | 1.24E-04 | 2.14E-02 | 1.139319418 |
| **TNFAIP8** | 1.4284181 | 8.149750757 | 3.757345964 | 7.21E-04 | 5.18E-02 | -0.457153115 |
| **SOSTDC1** | 1.185075141 | 8.317564616 | 2.423294591 | 2.15E-02 | 0.258710789 | -3.472092081 |
| **GATA3** | 1.108513314 | 8.216325527 | 3.313242531 | 0.002370163 | 0.090417879 | -1.529796515 |
| **GBP1** | 1.535847541 | 8.187850064 | 3.571368686 | 1.19E-03 | 6.54E-02 | -0.912556817 |
| **GBP2** | 1.615897665 | 8.402800421 | 5.69733536 | 3.00E-06 | 0.00242709 | 4.528720381 |
| **GEM** | 1.428033586 | 8.127939039 | 4.298260127 | 0.000160822 | 0.02398204 | 0.904970083 |
| **TSPAN13** | 1.073986319 | 9.56130702 | 2.786070883 | 9.06E-03 | 1.79E-01 | -2.721346942 |
| **DKK3** | 1.166336079 | 8.663287927 | 2.285473841 | 0.029346775 | 0.293079375 | -3.739410113 |
| **PYCARD** | 1.797360553 | 7.828540098 | 4.206120996 | 0.000208248 | 0.028900934 | 0.669827769 |
| **GZMA** | 1.184185591 | 6.774406243 | 2.619777367 | 0.013547286 | 0.209974234 | -3.073469228 |
| **ANXA1** | 1.005895067 | 11.53435709 | 3.502319732 | 1.44E-03 | 0.070172473 | -1.079480367 |
| **HBB** | 3.296744388 | 11.46394193 | 4.176401448 | 0.000226302 | 0.030196825 | 0.594210054 |
| **HCK** | 2.6398409 | 7.813158766 | 5.349869248 | 8.09E-06 | 0.004236103 | 3.628368393 |
| **HCLS1** | 1.736850227 | 9.718789727 | 6.039781163 | 1.14E-06 | 1.25E-03 | 5.409323565 |
| **AOAH** | 1.116127075 | 6.591542201 | 2.789684088 | 0.008979647 | 0.178911318 | -2.713555922 |
| **HLX** | 1.733114796 | 8.551807483 | 6.143738583 | 8.47E-07 | 0.001057373 | 5.674847263 |
| **HMOX1** | 1.229336838 | 8.989917666 | 2.462277565 | 0.019622014 | 0.248079788 | -3.394592999 |
| **IFI27** | 1.126270095 | 10.77401532 | 3.097420419 | 4.15E-03 | 0.12217618 | -2.030096345 |
| **IL10RA** | 1.758412442 | 7.741003398 | 3.760680306 | 7.14E-04 | 5.18E-02 | -0.448916736 |
| **IDO1** | 1.726049947 | 8.191623374 | 2.824352429 | 8.25E-03 | 1.70E-01 | -2.638510724 |
| **CXCL10** | 1.769454009 | 8.46969993 | 2.075231628 | 4.64E-02 | 3.53E-01 | -4.125882705 |
| **ISG20** | 1.45907201 | 7.652014498 | 3.10186306 | 0.004101815 | 0.121683825 | -2.019960048 |
| **ITGAM** | 1.287680606 | 7.088475734 | 2.706386842 | 0.011001205 | 0.19852981 | -2.891676554 |
| **ITGB2** | 2.464996223 | 7.840432579 | 4.523481362 | 8.52E-05 | 0.017435001 | 1.483545989 |
| **LCP1** | 1.51020218 | 8.874061162 | 3.429820859 | 1.74E-03 | 0.077903959 | -1.253364999 |
| **LTF** | 1.664651775 | 8.16718412 | 2.151985788 | 3.94E-02 | 3.34E-01 | -3.987900664 |
| **LYN** | 1.443538636 | 7.934231417 | 4.246664501 | 1.86E-04 | 2.64E-02 | 0.773168949 |
| **LYZ** | 3.461839278 | 9.138313494 | 5.513669632 | 5.07E-06 | 0.003398946 | 4.053439457 |
| **MEF2C** | 1.06367527 | 8.864186566 | 6.495995376 | 3.15E-07 | 0.000633354 | 6.566914021 |
| **MICB** | 1.283842867 | 8.432257502 | 3.832196515 | 5.87E-04 | 0.046662668 | -0.271701006 |
| **MNDA** | 1.944058451 | 7.68802875 | 3.405638692 | 0.001857523 | 0.079530964 | -1.311036019 |
| **MX1** | 1.053339525 | 10.15669734 | 2.129124163 | 0.041367918 | 0.340011054 | -4.029381896 |
| **MX2** | 1.208272091 | 8.90964076 | 2.784273318 | 9.10E-03 | 1.79E-01 | -2.725220815 |
| **NCF2** | 1.949044693 | 7.961713901 | 3.649872236 | 9.65E-04 | 5.98E-02 | -0.721313789 |
| **NME1** | 1.002868864 | 9.907840822 | 3.155207126 | 3.58E-03 | 1.14E-01 | -1.897698368 |
| **OAS1** | 1.883982044 | 9.238331718 | 3.726464874 | 7.84E-04 | 0.053795539 | -0.533320467 |
| **CALHM2** | 1.149877227 | 7.717842571 | 5.661573694 | 3.32E-06 | 0.00242709 | 4.436314132 |
| **HN1** | 1.223286865 | 7.92981769 | 4.344144204 | 1.41E-04 | 0.022918504 | 1.022435856 |
| **CKLF** | 1.029976095 | 9.114519974 | 3.542825412 | 1.29E-03 | 0.066954132 | -0.981709777 |
| **PLAC8** | 2.005619689 | 6.904337542 | 3.734320791 | 7.68E-04 | 5.36E-02 | -0.513963759 |
| **TNFRSF12A** | 1.505470167 | 8.862815372 | 3.067891809 | 0.004474091 | 0.126971151 | -2.097285314 |
| **CPVL** | 1.210937396 | 7.923576346 | 2.523069717 | 1.70E-02 | 0.229734218 | -3.27214045 |
| **PPP1R3C** | 1.081564064 | 9.810652913 | 2.790065265 | 0.008971252 | 0.178911318 | -2.712733669 |
| **GIMAP4** | 1.066095495 | 10.51388123 | 4.418183879 | 1.15E-04 | 2.05E-02 | 1.212445487 |
| **CYP26B1** | 1.486128163 | 7.889780569 | 2.303553239 | 0.028182476 | 0.288613415 | -3.704951498 |
| **C8orf4** | 1.464050458 | 9.91534729 | 5.851981163 | 1.93E-06 | 0.001946115 | 4.927420444 |
| **PSMB9** | 1.149756786 | 10.9382252 | 4.541310085 | 8.10E-05 | 0.017410612 | 1.529538356 |
| **TIGAR** | 1.062978409 | 7.552244523 | 3.320516087 | 0.002325317 | 0.089912261 | -1.512669443 |
| **PTGER2** | 1.262261601 | 7.121536319 | 3.228962396 | 0.002954177 | 0.102761217 | -1.727025988 |
| **KIAA1462** | 1.058038031 | 7.758668561 | 3.518102528 | 0.001376638 | 0.068968973 | -1.041436211 |
| **PTPRC** | 1.520083188 | 6.649678491 | 3.534597222 | 1.32E-03 | 0.067671768 | -1.001605512 |
| **PTPRE** | 1.045228874 | 7.86665182 | 2.953498611 | 5.98E-03 | 0.14881474 | -2.354464495 |
| **RAC2** | 1.298734215 | 9.039301916 | 3.527947469 | 1.34E-03 | 0.068020575 | -1.017671667 |
| **ACTA2** | 1.384026648 | 11.05579051 | 3.125026618 | 0.003865086 | 0.117698285 | -1.966994894 |
| **RNASE6** | 1.359659579 | 7.485663043 | 2.110629291 | 4.30E-02 | 0.3451199 | -4.062703594 |
| **S100A8** | 2.081494511 | 9.691964093 | 2.669478063 | 0.012026294 | 0.203903015 | -2.969582677 |
| **CCL2** | 1.345476319 | 7.561830542 | 2.208988717 | 3.48E-02 | 0.317231702 | -3.88308556 |
| **CCL4** | 1.868492407 | 8.8979264 | 3.486436536 | 1.50E-03 | 7.15E-02 | -1.117699022 |
| **MS4A6A** | 1.501160919 | 8.384973582 | 2.583967851 | 1.48E-02 | 2.18E-01 | -3.147577381 |
| **BMP2** | 1.278951698 | 9.788394794 | 2.523444183 | 1.70E-02 | 2.30E-01 | -3.271380245 |
| **TAGLN** | 1.463379584 | 9.351012226 | 2.813637808 | 0.008466152 | 0.172946727 | -2.661760242 |
| **TGFBI** | 1.792390756 | 9.264062891 | 2.736344105 | 0.010229685 | 0.191580633 | -2.827978933 |
| **THBD** | 1.075737036 | 7.978789754 | 3.271882779 | 2.64E-03 | 9.66E-02 | -1.626870472 |
| **TIE1** | 1.092945646 | 7.576198937 | 3.128567908 | 3.83E-03 | 0.117677204 | -1.958880539 |
| **TIMP1** | 1.022837412 | 11.80032286 | 2.73853 | 0.010175414 | 0.191367512 | -2.823315019 |
| **TLR1** | 1.020357579 | 5.710092232 | 2.467618682 | 0.019380496 | 0.24723966 | -3.383911837 |
| **TLR2** | 1.485748754 | 7.814631146 | 2.744684615 | 0.010024049 | 0.189405887 | -2.810171617 |
| **C1QA** | 2.063383904 | 7.751268347 | 2.483697284 | 0.018669798 | 0.242125823 | -3.351667305 |
| **TNFRSF1B** | 1.180125557 | 7.364571534 | 3.312878724 | 0.002372428 | 0.090417879 | -1.530652742 |
| **C3AR1** | 1.430225049 | 8.100369544 | 2.467368504 | 0.019391747 | 0.24723966 | -3.38441248 |
| **PHLDA2** | 1.558843075 | 6.458213679 | 2.657620994 | 0.012374019 | 0.205343598 | -2.994474172 |
| **TYROBP** | 2.98300807 | 8.71921773 | 5.200008138 | 1.24E-05 | 5.76E-03 | 3.238856823 |
| **UCP2** | 1.010563454 | 8.78271952 | 2.701769038 | 1.11E-02 | 0.19852981 | -2.90145851 |
| **LAPTM5** | 1.762819849 | 9.77238142 | 3.80520208 | 6.32E-04 | 4.86E-02 | -0.338716186 |
| **PXDN** | 1.040816746 | 9.163979004 | 2.955008217 | 5.96E-03 | 1.49E-01 | -2.351103791 |
| **CXCR4** | 1.349866177 | 8.55377937 | 3.111855793 | 0.003998045 | 0.120380038 | -1.997134603 |
| **LST1** | 1.573271773 | 7.782253429 | 4.647875154 | 5.99E-05 | 0.015716044 | 1.804910487 |
| **TFPI2** | 1.170968688 | 8.832996252 | 2.516368647 | 0.017298822 | 0.231815728 | -3.285732177 |
| **NETO2** | 1.723306281 | 8.011145364 | 4.053317625 | 3.19E-04 | 0.03535287 | 0.282354789 |
| **CASP1** | 1.320751283 | 7.350815488 | 4.341010088 | 0.000142605 | 0.022918504 | 1.014405063 |
| **GPR65** | 1.138480159 | 6.670757888 | 2.838605441 | 0.00796032 | 0.168017697 | -2.60750641 |
| **IFITM1** | 1.054065714 | 11.67585823 | 2.958870095 | 5.90E-03 | 0.148445424 | -2.342502292 |
| **ACTN1** | 1.243479948 | 7.358068609 | 3.104465594 | 0.004074545 | 0.121471738 | -2.014018802 |
| **TNFSF10** | 1.103006477 | 9.868178229 | 3.236999039 | 0.00289303 | 0.101838021 | -1.70831823 |
| **NMI** | 1.021717837 | 9.062323387 | 3.710262204 | 8.20E-04 | 0.055588936 | -0.573200209 |
| **CD14** | 1.928388565 | 9.036057353 | 4.099860707 | 0.000280191 | 0.033830252 | 0.400019583 |
| **LY86** | 1.034540159 | 8.018126254 | 3.002465142 | 5.28E-03 | 1.38E-01 | -2.244995501 |
| **CD36** | 1.289686314 | 5.894315327 | 2.084666815 | 0.045506152 | 0.35070011 | -4.109119423 |
| **ADAMTS1** | 1.925424756 | 9.230857747 | 4.673171181 | 5.57E-05 | 0.015189876 | 1.870383019 |
| **GMFG** | 1.07230973 | 9.241330034 | 4.63942951 | 6.13E-05 | 0.015754953 | 1.783059374 |
| **CYTIP** | 1.444773094 | 6.724741251 | 3.333208852 | 0.002249003 | 0.088451015 | -1.482742523 |
| **CD48** | 2.340554521 | 8.382224724 | 5.651865741 | 3.42E-06 | 2.43E-03 | 4.411217068 |
| **CD53** | 2.77291088 | 9.682215503 | 5.674505668 | 3.20E-06 | 0.00242709 | 4.469737939 |
| **ISG15** | 1.352320169 | 9.675726941 | 2.187461347 | 0.036446519 | 0.321695779 | -3.922899923 |
| **ADGRE5** | 1.071642431 | 8.333642554 | 4.331913553 | 0.000146302 | 0.022918504 | 0.991102239 |
| **RASSF2** | 1.312315694 | 7.500783902 | 3.410785842 | 0.001832353 | 0.079296895 | -1.298774908 |
| **RAPGEF5** | 1.082850875 | 8.278699721 | 4.128261748 | 2.59E-04 | 0.032314064 | 0.47197854 |
| **RIDA** | -1.404858899 | 9.11860268 | -2.65409705 | 0.012479151 | 0.205343598 | -3.001859091 |
| **MYL9** | -1.139408367 | 11.6116181 | -3.588297243 | 0.001140099 | 0.063729423 | -0.871445487 |
| **SLC19A2** | -1.260045943 | 6.687292907 | -6.131792338 | 8.76E-07 | 1.06E-03 | 5.64438292 |
| **LEFTY1** | -1.302868789 | 6.476979523 | -5.39819794 | 7.04E-06 | 4.24E-03 | 3.753875335 |
| **SLC17A3** | -1.598468896 | 9.363650571 | -2.070443493 | 4.69E-02 | 3.54E-01 | -4.134368143 |
| **FTCD** | -1.207557298 | 8.059013021 | -2.222774059 | 0.033728251 | 0.313740296 | -3.85744449 |
| **SLC27A2** | -1.496801272 | 9.109639244 | -2.100159255 | 0.044024236 | 0.348829764 | -4.08147278 |
| **ESM1** | -1.550234226 | 8.417158163 | -2.677732977 | 0.011789595 | 0.201911454 | -2.952213881 |
| **CHI3L1** | -1.0583532 | 11.87250355 | -2.278145677 | 0.029830943 | 0.294023513 | -3.753323769 |
| **CYP17A1** | -1.471979338 | 7.113312807 | -2.63447574 | 0.013079848 | 0.208071253 | -3.042869644 |
| **CYP27B1** | -2.858765526 | 7.768789338 | -6.734243395 | 1.62E-07 | 0.000603163 | 7.162654885 |
| **DEFB1** | -2.041889621 | 9.779850363 | -2.152239441 | 0.039351235 | 0.333920768 | -3.987438624 |
| **AFM** | -1.188476527 | 6.753612759 | -2.134319959 | 4.09E-02 | 0.338293425 | -4.019982653 |
| **DPEP1** | -1.399103764 | 8.820233201 | -2.095174335 | 4.45E-02 | 0.348829764 | -4.090384937 |
| **TSC22D3** | -1.120316741 | 11.825893 | -4.800217262 | 3.88E-05 | 0.012669398 | 2.199705199 |
| **EPHX1** | -1.307537254 | 8.239811227 | -4.053064339 | 0.000319154 | 0.03535287 | 0.281715373 |
| **ALB** | -2.51390987 | 6.880747528 | -3.150952781 | 0.00361557 | 0.114160332 | -1.907486053 |
| **FABP1** | -1.588334299 | 7.305360131 | -2.244624801 | 0.032138723 | 0.306200727 | -3.816570998 |
| **FKBP5** | -1.685801422 | 8.329460742 | -4.062403249 | 3.11E-04 | 3.52E-02 | 0.305297943 |
| **DIP2C** | -1.06748798 | 8.452447707 | -4.61651042 | 6.55E-05 | 0.015930989 | 1.723783688 |
| **FBXO21** | -1.168201217 | 10.41650496 | -4.769268614 | 4.24E-05 | 0.013126723 | 2.119414043 |
| **LPIN1** | -1.092325784 | 8.502391361 | -6.39617494 | 4.16E-07 | 0.000672562 | 6.315413351 |
| **TMEM2** | -1.447358504 | 8.973066213 | -6.578797509 | 2.50E-07 | 0.000603163 | 6.7747047 |
| **G6PC** | -1.907203988 | 5.479825589 | -3.451893251 | 0.0016428 | 0.075906542 | -1.20058006 |
| **SLC37A4** | -1.068172383 | 9.344609722 | -3.859063753 | 0.000545594 | 0.046662668 | -0.20485764 |
| **GHR** | -1.129292393 | 10.48695576 | -3.319613382 | 0.002330838 | 0.089912261 | -1.514795939 |
| **SLC6A16** | -1.244249347 | 7.273214099 | -4.539298976 | 8.15E-05 | 0.017410612 | 1.524349133 |
| **GSTA1** | -1.91350484 | 10.07005976 | -2.108123171 | 0.043279214 | 0.346154442 | -4.06720244 |
| **HPD** | -2.339718954 | 8.94215143 | -2.557555716 | 1.57E-02 | 0.22327652 | -3.201830932 |
| **IGF1** | -1.621566441 | 7.929502259 | -2.915883501 | 6.57E-03 | 1.56E-01 | -2.437907645 |
| **APOH** | -1.379341032 | 6.677843793 | -2.87131135 | 0.007340587 | 0.16322329 | -2.536033818 |
| **ARG2** | -1.130332916 | 7.23048032 | -3.38141245 | 0.00198053 | 0.083284359 | -1.368641971 |
| **LPL** | -1.935374001 | 8.951093054 | -3.488674417 | 0.001489349 | 0.071463652 | -1.112318292 |
| **MAOA** | -1.253876723 | 9.281233704 | -3.792143524 | 6.55E-04 | 4.90E-02 | -0.371081577 |
| **MT1X** | -1.107954285 | 9.510941421 | -2.538282184 | 0.016431826 | 0.225322304 | -3.241199913 |
| **PAH** | -1.567727035 | 10.72078849 | -2.090445225 | 0.044948342 | 0.349907335 | -4.09882534 |
| **PCK1** | -2.158054677 | 10.48775084 | -2.643313538 | 0.012806036 | 0.206711341 | -3.024420598 |
| **PDK4** | -2.133589177 | 7.967459341 | -7.701722928 | 1.16E-08 | 0.000140441 | 9.505992077 |
| **UPB1** | -1.219863602 | 7.307488702 | -2.042519372 | 0.049763103 | 0.364445691 | -4.183564219 |
| **CCDC91** | -1.050755989 | 10.13957593 | -3.89381653 | 0.00049585 | 0.045355275 | -0.118188979 |
| **PRODH2** | -1.354938654 | 10.04144547 | -2.401046922 | 0.022594524 | 0.264101178 | -3.515955012 |
| **SDC1** | -1.330188309 | 8.130051544 | -2.398532798 | 0.02272493 | 0.264917842 | -3.520894891 |
| **PBLD** | -1.136603119 | 9.008453816 | -2.323771175 | 0.026930046 | 0.282222869 | -3.666195874 |
| **XYLT1** | -1.111264775 | 7.302998736 | -4.933273142 | 0.0000266 | 0.011062871 | 2.545266893 |
| **USP46** | -1.049927684 | 9.258699673 | -3.511347888 | 0.001401755 | 0.069649353 | -1.057726284 |
| **SOWAHC** | -1.124288088 | 7.546180566 | -4.063409292 | 0.000310107 | 0.03521262 | 0.307839207 |
| **RASL11B** | -1.039689522 | 11.18406912 | -3.544628923 | 0.001282143 | 0.066954132 | -0.977346542 |
| **ELOVL4** | -1.093221906 | 6.305596762 | -3.472877129 | 0.00155348 | 0.073268407 | -1.150271838 |
| **UMOD** | -2.843265793 | 10.5106908 | -2.251940074 | 0.031621817 | 0.304467159 | -3.802824451 |
| **ZBTB16** | -2.264087666 | 7.881084284 | -3.367251839 | 0.002056022 | 0.084436766 | -1.402233356 |
| **ZNF189** | -1.418661523 | 9.172964451 | -7.022647205 | 7.31E-08 | 0.000441538 | 7.874548055 |
| **CACNB2** | -1.037091041 | 7.924522186 | -3.338797812 | 0.002216168 | 0.088199444 | -1.469549203 |
| **LRRC2** | -1.124154605 | 9.271682467 | -3.126568278 | 0.003849801 | 0.117677204 | -1.963462952 |
| **CALML3** | -1.126856678 | 6.62470469 | -3.079183611 | 0.004346923 | 0.125940929 | -2.071629931 |
| **C1orf21** | -1.449631569 | 8.554906677 | -3.850219787 | 0.000559011 | 0.046662668 | -0.226876282 |
| **USP9Y** | -1.111509719 | 4.868581731 | -2.728999324 | 0.010414026 | 0.192851143 | -2.843634102 |
| **TLN2** | -1.042106251 | 7.831024086 | -2.701344332 | 0.011136285 | 0.19852981 | -2.902357671 |
| **KCNK5** | -1.306860591 | 7.005931179 | -3.754627336 | 0.000726222 | 0.051884074 | -0.463866819 |
| **DLG5** | -1.218962185 | 9.549684511 | -4.840270591 | 0.0000346 | 0.012010107 | 2.303670969 |
| **SLC22A8** | -2.181565233 | 7.901843046 | -3.730706821 | 0.000775159 | 0.053788879 | -0.522870139 |
| **CXCL14** | -2.411662049 | 10.45026144 | -3.073477029 | 0.004410755 | 0.126603014 | -2.084601378 |
