# Supplementary material for: Periostin Contributes to Immunoglobulin a Nephropathy by Promoting the Proliferation of Mesangial Cells: A Weighted Gene Correlation Network Analysis
Source: Front Genet. 2021 Jan 7;11:595757. doi: 10.3389/fgene.2020.595757 (PMC7817997; doi:10.3389/fgene.2020.595757)
Supplement: Supplementary Table 6 — Significant GO terms for 37 genes upregulated in both GSE37460 and GSE104948. [file Table_6.DOCX]

| **Table S6** Significant GO terms for 37 genes upregulated in both GSE37460 and GSE104948 | | | | | |
| --- | --- | --- | --- | --- | --- |
| Category | ID | Description | Count | P.Value | Genes |
| Biological  process | GO:0030198 | extracellular matrix organization | 6 | 5.03E-05 | TGFBI, COL6A3, COL1A2, POSTN, ITGB2, FN1 |
|  | GO:0045087 | innate immune response | 6 | 0.001853253 | C1QA, NCF2, LY96, GATA3, PYCARD, TYROBP |
|  | GO:0007155 | cell adhesion | 6 | 0.002463176 | KIAA1462, TGFBI, COL6A3, POSTN, ITGB2, FN1 |
|  | GO:0006968 | cellular defense response | 3 | 0.007379305 | NCF2, LY96, TYROBP |
|  | GO:0008217 | regulation of blood pressure | 3 | 0.008085217 | ACTA2, COL1A2, HBB |
|  | GO:0006956 | complement activation | 3 | 0.014131915 | C1QA, FCN1, CFD |
|  | GO:0006508 | proteolysis | 5 | 0.019602804 | C1QA, FCN1, LTF, FGL2, CFD |
|  | GO:0071356 | cellular response to tumor necrosis factor | 3 | 0.021981645 | GATA3, PYCARD, POSTN |
|  | GO:0050900 | leukocyte migration | 3 | 0.026647035 | COL1A2, ITGB2, FN1 |
|  | GO:2000484 | positive regulation of interleukin-8 secretion | 2 | 0.026769395 | FCN1, PYCARD |
|  | GO:0045730 | respiratory burst | 2 | 0.026769395 | NCF2, CD52 |
|  | GO:0006915 | apoptotic process | 5 | 0.029433707 | C8ORF4, PYCARD, ITGB2, MECOM, PHLDA2 |
|  | GO:0051092 | positive regulation of NF-kappaB transcription factor activity | 3 | 0.031243712 | PYCARD, LTF, ITGB2 |
|  | GO:0034142 | toll-like receptor 4 signaling pathway | 2 | 0.036878844 | LY96, ITGB2 |
|  | GO:0051607 | defense response to virus | 3 | 0.046214288 | ISG15, PYCARD, IFI44L |
| Cellular component | GO:0005581 | collagen trimer | 4 | 7.88E-04 | C1QA, COL6A3, FCN1, COL1A2 |
|  | GO:0005576 | extracellular region | 11 | 8.42E-04 | C1QA, ISG15, TGFBI, COL6A3, FCN1, PYCARD, COL1A2, LTF, CFD, HBB, FN1 |
|  | GO:0005615 | extracellular space | 10 | 9.71E-04 | ACTA2, LY96, SOSTDC1, TGFBI, COL6A3, COL1A2, LTF, POSTN, CFD, FN1 |
|  | GO:0005578 | proteinaceous extracellular matrix | 5 | 0.001860425 | TGFBI, COL6A3, COL1A2, POSTN, FN1 |
|  | GO:0031012 | extracellular matrix | 5 | 0.002667776 | TGFBI, COL6A3, COL1A2, POSTN, FN1 |
|  | GO:0070062 | extracellular exosome | 12 | 0.016622706 | C1QA, ACTA2, TGFBI, COL6A3, COL1A2, LTF, FGL2, ITGB2, CD53, CFD, HBB, FN1 |
|  | GO:0005577 | fibrinogen complex | 2 | 0.017642759 | FGL2, FN1 |
| Molecular function | GO:0004252 | serine-type endopeptidase activity | 4 | 0.012231501 | C1QA, FCN1, LTF, CFD |
|  | GO:0008201 | heparin binding | 3 | 0.036775463 | LTF, POSTN, FN1 |

**Note:** Significant GO enrichment terms of DEGs with p<0.05 and count ≥2
