# Supplementary material for: Periostin Contributes to Immunoglobulin a Nephropathy by Promoting the Proliferation of Mesangial Cells: A Weighted Gene Correlation Network Analysis
Source: Front Genet. 2021 Jan 7;11:595757. doi: 10.3389/fgene.2020.595757 (PMC7817997; doi:10.3389/fgene.2020.595757)
Supplement: Supplementary Table 7 — Significant KEGG pathways for the 37 genes upregulated in both GSE37460 and GSE104948. [file Table_7.DOCX]

| ID | Description | Count | PValue |
| --- | --- | --- | --- |
| hsa05133 | Pertussis | 4 | 6.30E-04 |
| hsa05150 | Staphylococcus aureus infection | 3 | 0.006764598 |
| hsa04512 | ECM-receptor interaction | 3 | 0.016912655 |
| hsa05146 | Amoebiasis | 3 | 0.024521802 |
| hsa04151 | PI3K-Akt signaling pathway | 4 | 0.043071002 |

**Table S7** Significant KEGG pathways for the 37 genes upregulated in both GSE37460 and GSE104948

**Note:** Significant KEGG enrichment terms of DEGs with p<0.05 and count ≥2
