# Supplementary material for: Periostin Contributes to Immunoglobulin a Nephropathy by Promoting the Proliferation of Mesangial Cells: A Weighted Gene Correlation Network Analysis
Source: Front Genet. 2021 Jan 7;11:595757. doi: 10.3389/fgene.2020.595757 (PMC7817997; doi:10.3389/fgene.2020.595757)
Supplement: Supplementary Table 8 — Top 15 upregulated overlapping DEGs identified using the stress and betweenness algorithms in cytoHubba. [file Table_8.DOCX]

**Table S8** Top 15 upregulated overlapping DEGs identified using the stress and betweenness algorithms in CytoHubba

| **Gene symbol** | **Stress** | | **Betweenness** | |
| --- | --- | --- | --- | --- |
|  | **Rank** | **Score** | **Rank** | **Score** |
| FN1 | 1 | 646 | 1 | 173.14886 |
| ITGB2 | 2 | 372 | 2 | 70.68767 |
| C1QA | 3 | 286 | 3 | 47.64424 |
| TYROBP | 4 | 264 | 4 | 45.7034 |
| ACTA2 | 5 | 244 | 5 | 54.03261 |
| COL1A2 | 6 | 236 | 6 | 59.81399 |
| CD52 | 7 | 208 | 7 | 64.73333 |
| CD53 | 8 | 190 | 8 | 56.36696 |
| IL10RA | 9 | 186 | 9 | 37.30602 |
| TGFBI | 10 | 172 | 10 | 32.95317 |
| NCF2 | 11 | 168 | 11 | 47.70969 |
| ISG15 | 12 | 162 | 12 | 38.13771 |
| POSTN | 13 | 148 | 13 | 31.52121 |
| GATA3 | 14 | 130 | 14 | 32.96592 |
| FGL2 | 15 | 116 | 15 | 19.67001 |
