# Supplementary material for: Periostin Contributes to Immunoglobulin a Nephropathy by Promoting the Proliferation of Mesangial Cells: A Weighted Gene Correlation Network Analysis
Source: Front Genet. 2021 Jan 7;11:595757. doi: 10.3389/fgene.2020.595757 (PMC7817997; doi:10.3389/fgene.2020.595757)
Supplement: Supplementary Table 9 — Expression of 15 hub genes after eliminating the batch effect. [file Table_9.DOCX]

| **Table S9** Expression of 15 hub genes after eliminating the batch effect | | | | | | | | | | | | | | | |
| --- | --- | --- | --- | --- | --- | --- | --- | --- | --- | --- | --- | --- | --- | --- | --- |
| Gene symbol | ACTA2 | C1QA | CD52 | CD53 | COL1A2 | FGL2 | FN1 | GATA3 | IL10RA | ISG15 | ITGB2 | NCF2 | POSTN | TGFBI | TYROBP |
| GSM1046922 | 9.719055697 | 6.357100989 | 6.164900465 | 8.453968098 | 7.210711009 | 9.013953378 | 7.263575481 | 6.95394586 | 6.040538652 | 8.392495235 | 6.576360753 | 6.564779552 | 9.871667405 | 7.542784833 | 6.944592292 |
| GSM1046923 | 9.703513496 | 6.261391831 | 6.269245495 | 8.551660316 | 8.224680233 | 8.933447953 | 7.711713308 | 7.111348148 | 6.334603272 | 8.481769495 | 6.620413912 | 7.05988814 | 10.01858863 | 8.028259852 | 7.121213592 |
| GSM1046924 | 9.435499228 | 5.85145172 | 5.813004535 | 7.754917401 | 7.127625303 | 8.438863056 | 7.216291854 | 7.253507104 | 6.004885088 | 9.138135753 | 6.222151869 | 6.417413915 | 10.14107737 | 7.729379292 | 6.209792527 |
| GSM1046925 | 9.454033516 | 5.692377099 | 6.012786867 | 8.585198114 | 7.098664022 | 8.721392267 | 7.017573628 | 7.21181502 | 6.567704555 | 8.43582181 | 6.475796624 | 7.059102239 | 10.15004535 | 7.777815505 | 6.572846283 |
| GSM1046926 | 9.485048542 | 5.896989957 | 5.651163004 | 7.782042885 | 7.184086587 | 9.515998212 | 6.958925814 | 6.93407887 | 6.149559158 | 8.094021307 | 5.81054217 | 6.283345131 | 9.425801141 | 8.237695862 | 6.220832031 |
| GSM920350 | 10.07329241 | 6.374529459 | 6.234355502 | 8.629429293 | 7.870911267 | 9.524338709 | 7.830384527 | 6.648576596 | 6.327791981 | 9.230297861 | 6.668731642 | 6.628570273 | 9.838425658 | 8.483553335 | 7.275330802 |
| GSM920351 | 9.990630936 | 6.584281608 | 8.385218324 | 11.11409668 | 8.468728225 | 9.816391731 | 7.988131246 | 7.654516747 | 8.570635202 | 8.429820716 | 8.808835321 | 10.05844744 | 10.54623235 | 9.040868983 | 9.870666486 |
| GSM920352 | 10.37164975 | 7.0973311 | 5.647725586 | 8.704356282 | 8.295219863 | 6.914276887 | 7.858871031 | 6.258893087 | 7.056155848 | 8.404151943 | 6.87638935 | 6.708151251 | 8.275982728 | 9.032035917 | 7.427457441 |
| GSM920353 | 10.29137778 | 6.982773586 | 8.582060449 | 10.00966928 | 8.470840272 | 9.756998757 | 7.635521513 | 7.7621353 | 9.151869553 | 9.803117325 | 8.178682551 | 7.944507612 | 10.13688173 | 8.614411094 | 9.524926877 |
| GSM920401 | 10.09170116 | 6.289281311 | 6.535691581 | 8.137746492 | 8.639972853 | 9.849500024 | 7.857815715 | 8.152985099 | 6.195214681 | 9.057540798 | 6.245722842 | 7.133661002 | 10.34958394 | 8.902736622 | 7.204483231 |
| GSM920402 | 12.145575 | 6.840503448 | 7.13634914 | 9.756326279 | 7.882852089 | 10.79718222 | 7.628919392 | 9.04303101 | 7.782654652 | 9.962443069 | 7.353791439 | 8.218562116 | 11.70070419 | 8.706881515 | 8.807291656 |
| GSM920403 | 10.8549162 | 7.76776161 | 9.878433259 | 11.17498442 | 7.639411483 | 11.65187737 | 7.153351689 | 8.777058327 | 8.974403058 | 10.68706155 | 9.357831528 | 9.77007145 | 10.48581792 | 9.104531461 | 10.03641529 |
| GSM920404 | 11.38408445 | 8.002229654 | 7.775534757 | 10.11659194 | 8.666062853 | 9.86007917 | 8.183519936 | 8.30528187 | 8.082879276 | 9.273923667 | 8.330991129 | 8.095831779 | 11.60240839 | 9.580512785 | 8.81205163 |
| GSM920405 | 11.30820794 | 6.709585098 | 6.22743873 | 8.584335439 | 8.053877661 | 9.957668623 | 7.519244327 | 8.443208987 | 7.127683 | 10.43082658 | 6.793889181 | 7.352528948 | 10.72032799 | 8.284176074 | 7.835971735 |
| GSM920406 | 9.368932315 | 7.321044993 | 9.832227777 | 11.43425541 | 9.727140008 | 10.72410617 | 8.356608505 | 9.032268858 | 8.990887411 | 12.18672364 | 9.557840116 | 10.10258941 | 11.01904038 | 9.748825907 | 10.72444986 |
| GSM920407 | 12.3406434 | 8.410124536 | 6.886049872 | 9.851785577 | 10.9822751 | 11.10997522 | 9.799084015 | 8.226377891 | 8.069479298 | 9.414377991 | 8.590845147 | 7.788029173 | 11.77318145 | 10.00921622 | 8.847873092 |
| GSM920408 | 11.08348032 | 7.380400146 | 6.429898959 | 8.805075839 | 8.701842439 | 10.54623286 | 8.28267513 | 8.423365743 | 7.092312309 | 9.383230772 | 6.776784734 | 6.655287703 | 11.26432346 | 8.507236434 | 7.692378063 |
| GSM920409 | 11.1415718 | 10.73638901 | 8.532969723 | 10.9497029 | 11.89709345 | 10.35165946 | 10.37344154 | 6.666750391 | 8.978711269 | 8.262397412 | 9.565945944 | 9.611262906 | 12.63215529 | 12.30203838 | 10.1173368 |
| GSM920410 | 11.52356981 | 7.940974724 | 7.799492937 | 9.977909401 | 8.299202545 | 10.6542668 | 8.10153841 | 7.955645995 | 8.252499564 | 11.90738392 | 8.541798649 | 8.641866725 | 11.50745647 | 9.42668214 | 9.139745164 |
| GSM920411 | 11.12868769 | 7.02611953 | 7.115037537 | 9.884810116 | 7.67392601 | 10.42956905 | 7.343653425 | 8.581310245 | 7.105174732 | 9.54071198 | 7.884400552 | 7.787297944 | 11.49519094 | 8.342603675 | 8.377730196 |
| GSM920412 | 10.49406697 | 7.813543256 | 7.471271347 | 10.09925943 | 8.258413995 | 10.70802781 | 9.191344937 | 8.881688768 | 8.039515257 | 9.54273367 | 8.804718993 | 8.010453686 | 10.39904533 | 9.529532808 | 8.924976649 |
| GSM920413 | 11.39349084 | 8.701477034 | 6.802047469 | 9.316852566 | 10.47438384 | 10.33557347 | 9.503636891 | 7.798675103 | 7.295966693 | 9.393625646 | 7.826682497 | 7.531877322 | 11.14994208 | 8.567153164 | 8.53528815 |
| GSM920414 | 11.24025417 | 7.251056038 | 7.873548164 | 9.471541932 | 7.910594653 | 10.56329206 | 8.234357587 | 8.364557315 | 7.665392941 | 9.35770767 | 7.657654418 | 7.494777932 | 10.24570841 | 8.128351694 | 8.572749365 |
| GSM920415 | 10.41361561 | 7.276616938 | 8.410199464 | 9.756326279 | 8.203082685 | 10.62924244 | 8.413013699 | 8.192961492 | 7.88205167 | 10.04073878 | 8.209313345 | 7.843692413 | 11.53634781 | 8.783357406 | 8.775594005 |
| GSM920416 | 12.21714128 | 12.00778971 | 8.67597886 | 11.30983462 | 10.35333847 | 10.45879469 | 9.894063058 | 8.412422523 | 9.049912441 | 8.142732836 | 9.133314914 | 9.131029805 | 13.27177105 | 11.27092672 | 10.76810461 |
| GSM920417 | 11.82873888 | 10.92700586 | 9.060755381 | 11.25890664 | 9.623595443 | 10.78681605 | 9.038888272 | 8.243000503 | 9.330095909 | 9.319298508 | 9.138357362 | 9.300573458 | 12.40425216 | 10.32637812 | 10.22400476 |
| GSM920418 | 11.77663058 | 7.965424452 | 7.526526479 | 9.964295822 | 10.65543425 | 10.83131016 | 9.425405902 | 8.253040363 | 8.629817072 | 9.392995713 | 8.445330157 | 8.566725842 | 12.23518948 | 10.09619502 | 9.0350707 |
| GSM920419 | 12.10541622 | 8.118001244 | 8.050607515 | 10.27852754 | 10.91143868 | 10.0693098 | 10.10978214 | 8.623203186 | 8.689270784 | 9.425427405 | 8.768988722 | 8.540102849 | 12.13570281 | 10.28147711 | 9.348726662 |
| GSM920420 | 11.50127633 | 7.061452405 | 7.463126075 | 9.791915157 | 8.014907326 | 10.43225562 | 8.452018099 | 8.616177164 | 8.274204902 | 9.149640897 | 8.048034567 | 8.659417204 | 11.68601355 | 9.601276663 | 8.668354374 |
| GSM920421 | 10.98961066 | 6.992303508 | 6.924881347 | 9.185931483 | 8.249160228 | 10.22227622 | 7.665439234 | 8.245773907 | 7.386048369 | 9.54273367 | 7.349212574 | 7.374806545 | 10.99366447 | 8.669900146 | 7.82843454 |
| GSM920422 | 10.59196936 | 7.339975474 | 7.075473946 | 8.869067971 | 8.299658084 | 9.955238007 | 8.297511378 | 8.055148162 | 7.291953018 | 8.654215556 | 6.912443673 | 7.042857754 | 9.71578252 | 8.640567847 | 7.614454631 |
| GSM920423 | 10.66049796 | 8.772956126 | 7.107608719 | 9.950282389 | 7.83596897 | 10.90859228 | 7.580467549 | 8.75916923 | 7.554886141 | 12.86862383 | 7.462814978 | 7.730166388 | 10.74268928 | 7.972373266 | 8.518219282 |
| GSM920424 | 11.25859222 | 8.173075963 | 7.419844161 | 10.0753054 | 8.080086969 | 9.651376683 | 8.166978271 | 7.313943893 | 8.166133136 | 9.217378348 | 7.720510191 | 8.216413337 | 9.970627039 | 8.494304311 | 8.988586936 |
| GSM920425 | 10.7998069 | 6.174759418 | 7.532213097 | 9.28318181 | 7.313554677 | 10.46597766 | 6.783834914 | 8.117682826 | 7.279946988 | 9.97962842 | 7.269624596 | 7.933401258 | 10.08430257 | 7.403471438 | 8.339450081 |
| GSM920426 | 10.38716255 | 7.25947937 | 7.328400216 | 9.89764452 | 8.391306359 | 10.47060682 | 8.444090424 | 8.01361043 | 7.672233394 | 9.952619071 | 7.771229321 | 8.447239663 | 10.74427743 | 9.615702853 | 9.082977277 |
| GSM920427 | 12.145575 | 9.546396706 | 6.470476549 | 10.21961716 | 11.61520509 | 9.735034745 | 10.57602682 | 7.826031174 | 7.699817809 | 9.519342497 | 8.597751748 | 8.690803438 | 12.02264243 | 11.14169535 | 9.668991471 |
| GSM2810714 | 10.10027575 | 6.032370099 | 5.796081887 | 7.496695609 | 6.974390289 | 9.240825242 | 7.225499465 | 7.224250746 | 5.9846318 | 8.595563428 | 5.49753575 | 6.513156889 | 9.751072451 | 7.362296141 | 6.232616972 |
| GSM2810715 | 9.38035326 | 5.787803514 | 6.023850899 | 7.312834678 | 6.817670989 | 8.968098257 | 7.195129524 | 7.47832113 | 6.342611135 | 8.448935295 | 5.790970215 | 6.299562731 | 10.3509556 | 8.425433189 | 6.18886571 |
| GSM2810716 | 10.06379019 | 6.171124307 | 5.647735684 | 7.061169453 | 6.920058417 | 9.216225591 | 7.300398195 | 6.977140643 | 6.386826054 | 8.495853644 | 5.900902997 | 6.119653874 | 9.594482783 | 7.337269283 | 5.940370835 |
| GSM2810679 | 11.1539062 | 7.046031717 | 7.223509581 | 10.05245284 | 7.813352716 | 10.45337216 | 7.416824804 | 8.701176006 | 7.090060386 | 9.627888665 | 8.010031657 | 7.819408562 | 11.54714463 | 8.491029455 | 8.494814176 |
| GSM2810680 | 10.0640242 | 6.243818023 | 6.549398521 | 8.198698796 | 8.793553501 | 9.823390238 | 7.884613532 | 8.206837064 | 6.107289911 | 9.153612263 | 6.208167051 | 7.070195259 | 10.36629606 | 9.059432895 | 7.190029099 |
| GSM2810681 | 12.26102595 | 6.84053019 | 7.224858964 | 9.908687856 | 8.024644868 | 10.80858675 | 7.76688752 | 9.21098889 | 7.788873156 | 10.05441902 | 7.418542884 | 8.246803353 | 11.76431821 | 8.887895873 | 9.012660268 |
| GSM2810682 | 10.88877466 | 7.818106785 | 10.12351684 | 11.36259248 | 7.774868175 | 11.68315191 | 7.219734967 | 8.930351094 | 9.096596035 | 10.81418163 | 9.541601726 | 10.01989025 | 10.49978865 | 9.270849944 | 10.33173539 |
| GSM2810683 | 11.38241009 | 8.042564578 | 7.938691416 | 10.30470309 | 8.829155183 | 9.87034744 | 8.300980751 | 8.3746031 | 8.121287586 | 9.359765285 | 8.472548179 | 8.121693249 | 11.67351394 | 9.768593198 | 8.970008394 |
| GSM2810684 | 11.31089433 | 6.714862516 | 6.096168729 | 8.679462148 | 8.221463466 | 9.901815859 | 7.607659369 | 8.526689123 | 7.026846173 | 10.54206432 | 6.788258161 | 7.285408132 | 10.72273353 | 8.404252146 | 7.909231525 |
| GSM2810685 | 9.294003544 | 7.385377668 | 10.11824516 | 11.67896585 | 9.991120293 | 10.73480132 | 8.482039791 | 9.156896327 | 9.123104798 | 12.326116 | 9.784599272 | 10.35461238 | 11.10296531 | 9.949695082 | 11.13391363 |
| GSM2810686 | 12.48194832 | 8.515527985 | 6.941763261 | 10.03205385 | 11.1521284 | 11.18231426 | 9.958003188 | 8.305509576 | 8.119500818 | 9.52236124 | 8.740278565 | 7.775757391 | 11.90250863 | 10.21261895 | 9.047385129 |
| GSM2810687 | 11.11063884 | 7.420267887 | 6.425052553 | 8.873300564 | 8.876939143 | 10.55345754 | 8.395489644 | 8.509875721 | 7.022633642 | 9.476650774 | 6.788258161 | 6.539118538 | 11.31493839 | 8.654118779 | 7.741064113 |
| GSM2810688 | 11.16199047 | 10.91742435 | 8.699085064 | 11.18516186 | 12.11219954 | 10.36471047 | 10.58382005 | 6.526933478 | 9.171965313 | 8.33601877 | 9.777579138 | 9.798702669 | 12.75816912 | 12.50571315 | 10.46999203 |
| GSM2810689 | 11.54929064 | 8.025141084 | 7.899111853 | 10.14723444 | 8.467914846 | 10.69645477 | 8.182561132 | 7.984907813 | 8.340996368 | 12.07633364 | 8.682135288 | 8.738861533 | 11.57378508 | 9.610184342 | 9.369455937 |
| GSM2810690 | 11.58528226 | 7.11914004 | 7.565809325 | 9.947343734 | 8.222412568 | 10.48081915 | 8.55175026 | 8.738525819 | 8.360975859 | 9.206911603 | 8.175443262 | 8.770585334 | 11.74940307 | 9.762364068 | 8.847739957 |
| GSM2810691 | 10.5051741 | 7.914818819 | 7.568269377 | 10.23536572 | 8.44624254 | 10.715731 | 9.311174172 | 9.023854176 | 8.091989649 | 9.634682514 | 8.954135949 | 8.040986041 | 10.43369809 | 9.707741208 | 9.112719653 |
| GSM2810692 | 11.45631712 | 8.800045886 | 6.816094878 | 9.47375573 | 10.64280845 | 10.32133376 | 9.637275915 | 7.748131662 | 7.313177149 | 9.531413435 | 7.907048501 | 7.504070922 | 11.18422678 | 8.720189131 | 8.671125875 |
| GSM2810693 | 11.2622319 | 7.296793257 | 8.041791847 | 9.60716421 | 8.077507164 | 10.59805409 | 8.344777507 | 8.426336645 | 7.674548313 | 9.451191167 | 7.734134121 | 7.472362156 | 10.24440534 | 8.307755306 | 8.754308944 |
| GSM2810694 | 10.39978555 | 7.325369634 | 8.595666014 | 9.918386327 | 8.352011856 | 10.66469413 | 8.557181874 | 8.240499194 | 7.912133854 | 10.1499022 | 8.316321638 | 7.814524354 | 11.67637095 | 8.952672718 | 8.944853621 |
| GSM2810695 | 12.40638112 | 12.24074917 | 8.93011317 | 11.52300027 | 10.57637825 | 10.43431952 | 9.76711542 | 8.504299057 | 9.3720671 | 8.279426463 | 9.33522924 | 9.294462533 | 13.4466839 | 11.54480654 | 11.2158321 |
| GSM2810696 | 11.88509119 | 11.08992706 | 9.283892157 | 11.47820209 | 9.793166921 | 10.8129056 | 9.109229085 | 8.360228201 | 9.484798994 | 9.376435066 | 9.314953376 | 9.466545431 | 12.591294 | 10.56777197 | 10.61046245 |
| GSM2810697 | 11.87447257 | 7.967388977 | 7.582241631 | 10.11671396 | 10.83108316 | 10.83376149 | 9.557708146 | 8.297876131 | 8.798034284 | 9.486231666 | 8.595467563 | 8.655779877 | 12.36306862 | 10.28252352 | 9.232435867 |
| GSM2810698 | 12.20083641 | 8.202293202 | 8.151049899 | 10.42671802 | 11.06563472 | 10.05885637 | 10.25122756 | 8.728629522 | 8.790209859 | 9.514505048 | 8.897115854 | 8.589995915 | 12.27035638 | 10.47803132 | 9.586491075 |
| GSM2810699 | 11.01105109 | 7.033349991 | 6.959217342 | 9.31709412 | 8.449922407 | 10.18770217 | 7.725789328 | 8.310116727 | 7.379998208 | 9.647358359 | 7.400921542 | 7.419405631 | 11.01421418 | 8.86621854 | 7.897990326 |
| GSM2810700 | 10.60351133 | 7.404651296 | 7.170657925 | 9.001653798 | 8.490172811 | 9.939449009 | 8.437397701 | 8.105365917 | 7.233361143 | 8.747310984 | 6.914149856 | 6.932716772 | 9.705612723 | 8.82440564 | 7.66609542 |
| GSM2810701 | 10.66227199 | 8.833881834 | 7.16317789 | 10.11671396 | 8.012556414 | 10.94994921 | 7.684843849 | 8.886263751 | 7.634508574 | 12.95512127 | 7.524048482 | 7.73733121 | 10.76544654 | 8.125557931 | 8.659018583 |
| GSM2810702 | 11.30490242 | 8.232485868 | 7.537073766 | 10.23981394 | 8.237209072 | 9.643237183 | 8.281658276 | 7.145345591 | 8.247022502 | 9.311975676 | 7.848545726 | 8.300304127 | 9.920353941 | 8.669175876 | 9.218691506 |
| GSM2810703 | 10.81813113 | 6.122843973 | 7.657731155 | 9.429887599 | 7.44326134 | 10.4851692 | 6.777962417 | 8.223893826 | 7.26758127 | 10.06761861 | 7.354051792 | 7.934356346 | 10.10313966 | 7.515876529 | 8.475949348 |
| GSM2810704 | 10.40166035 | 7.310088091 | 7.438644736 | 10.02789965 | 8.554280134 | 10.44253501 | 8.622398675 | 8.06101657 | 7.699025006 | 10.07496195 | 7.840963504 | 8.575927631 | 10.80482554 | 9.798977324 | 9.312996623 |
| GSM2810705 | 12.22153955 | 9.629518363 | 6.449114714 | 10.42348086 | 11.82227614 | 9.704631797 | 10.78588829 | 7.839313997 | 7.768372777 | 9.593388744 | 8.76059018 | 8.777323797 | 12.15175622 | 11.36380415 | 9.958689199 |
