# Supplementary material for: Periostin Contributes to Immunoglobulin a Nephropathy by Promoting the Proliferation of Mesangial Cells: A Weighted Gene Correlation Network Analysis
Source: Front Genet. 2021 Jan 7;11:595757. doi: 10.3389/fgene.2020.595757 (PMC7817997; doi:10.3389/fgene.2020.595757)
Supplement: Supplementary Table 10 — GS and MM of genes and clinical features of IgAN in the royalblue module. [file Table_10.DOCX]

**Table S10** GS and MM of genes and clinical features of IgAN in the royalblue module

| **Gene symbol** | **GS.Creatinine** | **p.GS.Creatinine** | **GS.eGFR** | **p.GS.eGFR** | **MMroyalblue** | **p.MMroyalblue** |
| --- | --- | --- | --- | --- | --- | --- |
| **ADAM12** | 0.413183123 | 0.099257534 | -0.251165194 | 0.330846939 | 0.685916142 | 0.002366459 |
| **AGTR1** | 0.61623381 | 0.00843319 | -0.509849846 | 0.036548417 | 0.949769523 | 5.73E-09 |
| **ARSK** | 0.458470817 | 0.064181124 | -0.520273927 | 0.032276208 | 0.660016369 | 0.003936456 |
| **ATF3** | -0.330766974 | 0.1947076 | 0.328035321 | 0.198642662 | -0.562158814 | 0.018834812 |
| **ATP1A2** | 0.515003987 | 0.034385322 | -0.489079377 | 0.046331361 | 0.791814139 | 0.000152036 |
| **BAZ2B** | 0.279292817 | 0.277643504 | -0.093632365 | 0.720764652 | 0.750548137 | 0.000518051 |
| **BHLHE40** | -0.381730669 | 0.130541622 | 0.376373837 | 0.136481808 | -0.584411514 | 0.013752758 |
| **C10orf10** | -0.122921012 | 0.638353943 | 0.101679963 | 0.6977844 | -0.58043804 | 0.014569952 |
| **C2CD4A** | -0.313725116 | 0.220104976 | 0.40324781 | 0.108491333 | -0.487527609 | 0.047134253 |
| **C8orf82** | -0.154090155 | 0.5548678 | -0.035316885 | 0.892955996 | -0.716193302 | 0.00122108 |
| **CCDC66** | -0.069442839 | 0.79114012 | 0.288547161 | 0.261356379 | 0.461696596 | 0.062091969 |
| **CCDC8** | -0.504544804 | 0.038881592 | 0.41176096 | 0.100543582 | -0.812307173 | 7.45E-05 |
| **CCM2** | -0.18889474 | 0.46778602 | 0.154021493 | 0.555046313 | -0.723975201 | 0.001016478 |
| **CD33** | -0.273516132 | 0.28811667 | 0.164226661 | 0.528794639 | -0.592131933 | 0.012268608 |
| **CDH13** | 0.021536524 | 0.934612903 | -0.039658347 | 0.879881815 | 0.569011409 | 0.01713479 |
| **CFB** | -0.501075494 | 0.040467499 | 0.455512185 | 0.066142507 | -0.863913025 | 7.83E-06 |
| **CH25H** | -0.475962243 | 0.053452083 | 0.508738387 | 0.037028168 | -0.804215152 | 9.97E-05 |
| **CNGA1** | 0.456850405 | 0.065249954 | -0.441894933 | 0.075743357 | 0.737424162 | 0.000729744 |
| **COL4A1** | 0.422632737 | 0.091009341 | -0.538428483 | 0.025761141 | 0.600420208 | 0.010819519 |
| **COL4A2** | 0.336251071 | 0.186963029 | -0.49008098 | 0.045818626 | 0.497420009 | 0.042191092 |
| **COL5A2** | 0.403125682 | 0.10860849 | -0.308013471 | 0.22907125 | 0.600583471 | 0.010792407 |
| **CPTP** | -0.176856074 | 0.497114304 | 0.104577962 | 0.689568313 | -0.527563557 | 0.029523935 |
| **CYSLTR1** | 0.272754638 | 0.289514814 | -0.024501424 | 0.925633144 | 0.661542828 | 0.003825127 |
| **DAAM2** | 0.452748504 | 0.068014244 | -0.354754696 | 0.162347742 | 0.835598019 | 2.96E-05 |
| **DUSP4** | 0.652845678 | 0.004495166 | -0.713134399 | 0.001310277 | 0.518892719 | 0.032819135 |
| **EBF1** | 0.473940946 | 0.054618186 | -0.325327664 | 0.20259418 | 0.840304857 | 2.42E-05 |
| **EMILIN1** | -0.138779281 | 0.595280649 | 0.168760468 | 0.517316912 | -0.733896231 | 0.000797496 |
| **ERMAP** | 0.45449689 | 0.066825659 | -0.464182704 | 0.060516541 | 0.813728446 | 7.07E-05 |
| **FAM107A** | -0.310200589 | 0.22561079 | 0.110417797 | 0.673111893 | -0.75470255 | 0.000462821 |
| **FAP** | -0.362166554 | 0.153133778 | 0.358083347 | 0.158164587 | -0.630468315 | 0.006663767 |
| **FGD1** | 0.081808115 | 0.754938207 | -0.040179271 | 0.878314973 | 0.523141261 | 0.031171113 |
| **FRZB** | 0.387451864 | 0.12439832 | -0.21588988 | 0.405286813 | 0.719948601 | 0.001118481 |
| **GATA3** | 0.276023226 | 0.283542386 | -0.078849474 | 0.763559427 | 0.877088343 | 3.80E-06 |
| **GLIPR1** | -0.444459085 | 0.073862141 | 0.515212743 | 0.034299822 | -0.77924761 | 0.000226844 |
| **GLT8D2** | 0.223231381 | 0.389103837 | -0.020105594 | 0.938949474 | 0.644879871 | 0.00518933 |
| **GPR183** | -0.297698851 | 0.245845058 | 0.321650687 | 0.20804188 | -0.814268948 | 6.93E-05 |
| **GPX2** | -0.428255176 | 0.086342457 | 0.310630837 | 0.224934008 | -0.731322147 | 0.000850149 |
| **GTF2E1** | 0.383499323 | 0.128620437 | -0.403710953 | 0.108047854 | 0.700001881 | 0.001756455 |
| **HCG11** | 0.375492868 | 0.137476291 | -0.4265906 | 0.087705692 | 0.737726823 | 0.000724161 |
| **HEATR5B** | 0.455030156 | 0.0664662 | -0.464252519 | 0.060472732 | 0.660063521 | 0.003932978 |
| **HS3ST3A1** | 0.552313429 | 0.021504985 | -0.423331428 | 0.090419731 | 0.79464879 | 0.000138402 |
| **IGFLR1** | -0.172761592 | 0.507284765 | 0.132231878 | 0.612920635 | -0.61676314 | 0.008361294 |
| **IGLVI-70** | -0.209031679 | 0.420723949 | 0.177021321 | 0.496705894 | -0.44850347 | 0.070964751 |
| **IL32** | 0.053151992 | 0.839447075 | -0.304091122 | 0.235361488 | -0.366519413 | 0.147891953 |
| **IMPDH2** | -0.475426758 | 0.053759181 | 0.467263107 | 0.05860587 | -0.752651145 | 0.000489446 |
| **LHPP** | -0.373183085 | 0.140107419 | 0.274770478 | 0.285822532 | -0.581899078 | 0.01426514 |
| **LIAS** | 0.535771351 | 0.026645044 | -0.492443958 | 0.044625935 | 0.733418445 | 0.00080706 |
| **LINC00641** | 0.203790308 | 0.432726824 | -0.049896529 | 0.849171391 | 0.510353248 | 0.036332693 |
| **LINC01220** | -0.174028587 | 0.504127201 | 0.204919282 | 0.430126564 | -0.473898002 | 0.054643165 |
| **LIPG** | -0.250430733 | 0.332310232 | 0.322160051 | 0.207281605 | -0.531714949 | 0.028039382 |
| **LOC100506100** | 0.311859865 | 0.223007903 | -0.201037395 | 0.439101374 | 0.578433947 | 0.014996383 |
| **LOC101060391** | 0.263749245 | 0.30635856 | -0.038192479 | 0.884293115 | 0.461240033 | 0.062384557 |
| **LOC101929165** | 0.28303934 | 0.270976943 | -0.112393438 | 0.66757578 | 0.484735874 | 0.048605013 |
| **MIR155HG** | -0.401726095 | 0.109957513 | 0.441776555 | 0.075831043 | -0.610742165 | 0.009208644 |
| **NABP2** | -0.284039347 | 0.269214293 | 0.125114158 | 0.63232745 | -0.734656365 | 0.000782475 |
| **NDC80** | 0.181863759 | 0.48480935 | -0.181854587 | 0.484831752 | 0.620816818 | 0.007826798 |
| **NOP14** | -0.449053799 | 0.07057704 | 0.425376748 | 0.088709547 | -0.562940576 | 0.018634464 |
| **NSG1** | 0.406174261 | 0.105710703 | -0.295697066 | 0.249187354 | 0.700387454 | 0.001741781 |
| **NSUN7** | 0.432482869 | 0.082949057 | -0.34170834 | 0.179461346 | 0.78184717 | 0.000209264 |
| **NUDT10** | 0.449193224 | 0.070479061 | -0.304194903 | 0.23519366 | 0.895938416 | 1.15E-06 |
| **OR7E12P** | -0.325676135 | 0.202082774 | 0.300528815 | 0.241168252 | -0.743158102 | 0.00062983 |
| **PCDH17** | 0.376672081 | 0.136146262 | -0.427564536 | 0.086906197 | 0.523494193 | 0.031037123 |
| **PDGFD** | 0.479848282 | 0.051262589 | -0.430379177 | 0.084625293 | 0.724901511 | 0.000994135 |
| **PIM1** | -0.311594371 | 0.223423082 | 0.165836506 | 0.524705925 | -0.80054627 | 0.000113299 |
| **PLA2G15** | -0.126781953 | 0.627759134 | 0.007407349 | 0.977490599 | -0.478247494 | 0.052156222 |
| **POSTN** | 0.659533386 | 0.003972223 | -0.671603413 | 0.003153586 | 0.820126523 | 5.55E-05 |
| **PSMD5-AS1** | -0.535925646 | 0.026593086 | 0.571951151 | 0.016443482 | -0.617863159 | 0.008213451 |
| **PYGO1** | 0.217776491 | 0.401094156 | 0.025681173 | 0.922062354 | 0.532195275 | 0.027871403 |
| **PYROXD2** | 0.389288415 | 0.122469831 | -0.428388376 | 0.086234036 | 0.39976476 | 0.11186787 |
| **RAPH1** | 0.411399408 | 0.100872417 | -0.33397051 | 0.190158448 | 0.832141809 | 3.43E-05 |
| **RGS1** | -0.422702878 | 0.090950026 | 0.489241935 | 0.046247853 | -0.687775933 | 0.002277201 |
| **RPPH1** | -0.212978153 | 0.411803404 | 0.292198367 | 0.25509695 | -0.39500729 | 0.116598804 |
| **S1PR3** | 0.274651756 | 0.286039193 | -0.025230288 | 0.923426904 | 0.689006858 | 0.002219653 |
| **SAMD1** | 0.331703729 | 0.193370044 | -0.403789167 | 0.107973088 | 0.410810279 | 0.101409876 |
| **SDCCAG3** | -0.607162218 | 0.009744047 | 0.598577119 | 0.011129351 | -0.575056387 | 0.015737243 |
| **SDPR** | 0.410563386 | 0.10163572 | -0.469863465 | 0.057028159 | 0.764896239 | 0.000347705 |
| **SLC35E2** | 0.549521711 | 0.0223133 | -0.49780679 | 0.042006138 | 0.651080103 | 0.004642132 |
| **SLCO4A1** | -0.195009778 | 0.453225735 | 0.215071705 | 0.407112327 | -0.696730132 | 0.001885104 |
| **SLF1** | 0.513276863 | 0.035098962 | -0.4761656 | 0.053335804 | 0.700684563 | 0.001730542 |
| **SLPI** | -0.637721091 | 0.005884677 | 0.696838531 | 0.001880721 | -0.69868527 | 0.00180733 |
| **SPRY3** | 0.328186706 | 0.198423233 | -0.140976001 | 0.589409183 | 0.684853294 | 0.00241874 |
| **SRGAP3** | -0.358669852 | 0.157435169 | 0.312029587 | 0.222742752 | -0.677768335 | 0.002791885 |
| **ST3GAL5** | -0.58166795 | 0.014313022 | 0.54988972 | 0.022205416 | -0.642429277 | 0.005419459 |
| **STK17B** | 0.708372039 | 0.00145978 | -0.642950573 | 0.005369829 | 0.844768183 | 1.98E-05 |
| **SYNPO2** | 0.516329009 | 0.033845388 | -0.367672739 | 0.146523925 | 0.867125331 | 6.61E-06 |
| **TAC1** | -0.322547369 | 0.206704707 | 0.420599389 | 0.092740978 | -0.583977964 | 0.013840122 |
| **TCIRG1** | 0.200043724 | 0.441414085 | -0.362871134 | 0.152276843 | -0.407206309 | 0.104742263 |
| **THBD** | -0.266207908 | 0.30170332 | 0.180396368 | 0.488399648 | -0.451362496 | 0.068967475 |
| **TMIE** | -0.350699821 | 0.167543714 | 0.156290811 | 0.54915979 | -0.6776327 | 0.002799461 |
| **TP53I3** | -0.059370953 | 0.820932571 | -0.200273256 | 0.44087931 | -0.350561018 | 0.167723534 |
| **TPM4** | 0.438277245 | 0.078456692 | -0.357114079 | 0.159375054 | 0.581875956 | 0.014269925 |
| **TTC32** | 0.376810016 | 0.13599127 | -0.153868894 | 0.555443137 | 0.729325885 | 0.000892935 |
| **TTR** | 0.612299145 | 0.008983238 | -0.516886009 | 0.033620362 | 0.759835055 | 0.000401438 |
| **VCAM1** | -0.374176107 | 0.13897203 | 0.447044958 | 0.07199984 | -0.457829877 | 0.064602333 |
| **WISP1** | 0.459344148 | 0.063610463 | -0.34230504 | 0.178653453 | 0.883273005 | 2.63E-06 |
| **ZC4H2** | -0.319086244 | 0.211897041 | 0.285892409 | 0.265966697 | -0.412344677 | 0.100014303 |
| **ZNF18** | -0.199166298 | 0.443461428 | 0.007404125 | 0.977500391 | -0.563457488 | 0.01850291 |
| **ZNF573** | -0.393800438 | 0.11782095 | 0.265915539 | 0.302254669 | -0.405870475 | 0.105996973 |
| **ZNF706** | 0.168856199 | 0.517075814 | 0.020305193 | 0.938344471 | 0.607147755 | 0.009746259 |
| **ZNF782** | -0.121050002 | 0.643512239 | 0.355521954 | 0.161376961 | 0.321476915 | 0.208301665 |
| **ZNF814** | 0.395657412 | 0.115944151 | -0.199889638 | 0.44177327 | 0.582831605 | 0.014073235 |
